# Impact of Prosigna test on adjuvant treatment decision in lymph node-negative early breast cancer—a prospective national multicentre study (EMIT-1)

**DOI:** 10.1016/j.esmoop.2024.103475

**Published:** 2024-06-04

**Authors:** H.O. Ohnstad, E.S. Blix, L.A. Akslen, B. Gilje, S.X. Raj, H. Skjerven, E. Borgen, E.A.M. Janssen, E. Mortensen, M.B. Brekke, R.S. Falk, E. Schlichting, B. Boge, S. Songe-Møller, P. Olsson, A. Heie, B. Mannsåker, M.A. Vestlid, T. Kursetgjerde, B. Gravdehaug, P. Suhrke, E. Sanchez, J. Bublevic, O.D. Røe, G.A. Geitvik, E.H. Halset, M.C. Rypdal, A. Langerød, J. Lømo, Ø. Garred, A. Porojnicu, O. Engebraaten, J. Geisler, M. Lyngra, M.H. Hansen, H. Søiland, T. Nakken, L. Asphaug, V. Kristensen, T. Sørlie, J.F. Nygård, C.E. Kiserud, K.V. Reinertsen, H.G. Russnes, B. Naume

**Affiliations:** 1 Department of Oncology, Division of Cancer Medicine, Oslo University Hospital, Oslo; 2 Department of Oncology, University of North Norway, Tromsø; 3 Department of Clinical Medicine, UiT The Arctic University of Norway, Tromsø; 4 Centre for Cancer Biomarkers CCBIO, Department of Clinical Medicine, Section for Pathology, University of Bergen, Bergen; 5 Department of Pathology Haukeland University Hospital, Bergen; 6 Department of Haematology and Oncology, Stavanger University Hospital, Stavanger; 7 Department of Oncology, St Olavs Hospital, Trondheim; 8 Department of Breast Surgery, Vestre Viken Hospital Trust, Drammen; 9 Department of Pathology, Division of Laboratory Medicine, Oslo University Hospital, Oslo; 10 Department of Pathology, Stavanger University Hospital, Stavanger; 11 Department of Chemistry, Bioscience and Environmental Engineering, Stavanger University, Stavanger, Norway; 12 Menzies Health Institute Queensland and Griffith University, Southport, Australia; 13 Department of Pathology, University of North Norway, Tromsø; 14 Department of Pathology, St Olavs Hospital, Trondheim; 15 Oslo Centre for Biostatistics and Epidemiology, Oslo University Hospital, Oslo; 16 Department of Oncology, Breast and Endocrine Surgery Unit, Division of Cancer Medicine, Oslo University Hospital, Oslo; 17 Department of Oncology, Hospital of Southern Norway, Kristiansand; 18 Department of Oncology, Østfold Hospital Trust, Kalnes; 19 Department of Breast Surgery, Innlandet Hospital Trust, Hamar; 20 Department of Breast Surgery, Haukeland University Hospital, Bergen; 21 Department of Oncology, Nordland Hospital, Bodø; 22 Department of Breast Surgery, Telemark Hospital Trust, Skien; 23 Department of Oncology, Møre og Romsdal Hospital Trust, Ålesund; 24 Department of Breast Surgery, Akershus University Hospital, Lørenskog; 25 Department of Pathology, Vestfold Hospital Trust, Tønsberg; 26 Department of Oncology, Haugesund Hospital, Haugesund; 27 Department of Oncology, Førde Central Hospital, Førde; 28 Department of Oncology, Levanger Hospital, Levanger; 29 Department of Cancer Genetics, Institute for Cancer Research, Oslo University Hospital, Oslo; 30 Department of Oncology, Vestre Viken Hospital Trust, Drammen; 31 Institute of Clinical Medicine, University of Oslo, Oslo; 32 Department of Oncology, Akershus University Hospital, Lørenskog; 33 Department of Pathology, Akershus University Hospital, Lørenskog; 34 Department of Breast Surgery, University of North Norway, Tromsø; 35 Department of Research, Stavanger University Hospital, Stavanger; 36 Department of Clinical Science, University of Bergen, Bergen; 37 User representative, Oslo University Hospital, Oslo; 38 Clinical Trials Unit, Oslo University Hospital, Oslo; 39 Institute of Health and Society, Faculty of Medicine, University of Oslo, Oslo; 40 Cancer Registry of Norway, Oslo; 41 National Advisory Unit for Late Effects after Cancer Treatment, Oslo University Hospital, Oslo, Norway

**Keywords:** early breast cancer, Prosigna, adjuvant treatment, chemotherapy, endocrine treatment, decision impact

## Abstract

**Background:**

EMIT-1 is a national, observational, single-arm trial designed to assess the value of the Prosigna, Prediction Analysis of Microarray using the 50 gene classifier (PAM50)/Risk of Recurrence (ROR), test as a routine diagnostic tool, examining its impact on adjuvant treatment decisions, clinical outcomes, side-effects and cost-effectiveness. Here we present the impact on treatment decisions.

**Patients and methods:**

Patients with hormone receptor-positive, human epidermal growth factor receptor 2-negative pT1-pT2 lymph node-negative early breast cancer (EBC) were included. The Prosigna test and standard histopathology assessments were carried out. Clinicians’ treatment decisions were recorded before (pre-Prosigna) and after (post-Prosigna) the Prosigna test results were disclosed.

**Results:**

Of 2217 patients included, 2178 had conclusive Prosigna results. The pre-Prosigna treatment decisions were: no systemic treatment (NT) in 27% of patients, endocrine treatment alone (ET) in 38% and chemotherapy (CT) followed by ET (CT + ET) in 35%. Post-Prosigna treatment decisions were 25% NT, 51% ET and 24% CT + ET, respectively. Adjuvant treatment changed in 28% of patients, including 21% change in CT use. Among patients assigned to CT + ET pre-Prosigna, 45% were de-escalated to ET post-Prosigna. Of patients assigned to ET, 12% were escalated to CT + ET and 8% were de-escalated to NT; of those assigned to NT, 18% were escalated to ET/CT + ET. CT was more frequently recommended for patients aged ≤50 years. In the subgroup with pT1c-pT2 G2 and intermediate Ki67 (0.5-1.5× local laboratory median Ki67 score), the pre-Prosigna CT treatment decision varied widely across hospitals (3%-51%). Post-Prosigna, the variability of CT use was markedly reduced (8%-24%). The correlation between Ki67 and ROR score within this subgroup was poor (*r* = 0.25-0.39). The median ROR score increased by increasing histological grade, but the ROR score ranges were wide (for G1 0-79, G2 0-90, G3 16-94).

**Conclusion:**

The Prosigna test result changed adjuvant treatment decisions in all EBC clinical risk groups, markedly decreased the CT use for patients categorized as higher clinical risk pre-Prosigna and reduced treatment decision discrepancies between hospitals.

## Highlights

• Forty-five percent of patients assigned to CT by routine classification were de-escalated to no CT by the Prosigna test.
• The Prosigna test identified lower-risk patients with molecular higher risk who were offered escalated systemic treatment.
• Incorporating the Prosigna test in the diagnostic work-up significantly reduced the treatment discrepancies between hospitals.
• The correlations between Ki67 and ROR score among patients within different treatment/risk groups were poor.

## Introduction

The hormone receptor (HR)-positive, human epidermal growth factor receptor 2 (HER2)-negative subtype is the most common of all breast cancer subclasses. The majority of patients with HR+/HER2−breast cancer are diagnosed in the early stage of the disease with no evidence of axillary lymph node metastasis (pN0) and overall good prognosis.^1^, ^2^, ^3^ However, the risk of relapse varies substantially depending on the individual biology of the disease.^4^ Stage and primary tumour factors are used for risk classification and support for adjuvant systemic treatment decisions.^5^^,^^6^ A large number of patients with early-stage breast cancer (EBC) with relatively small primary tumours and pN0 receive unnecessary treatment with chemotherapy.^4^ Conversely, subgroups of patients considered as clinically low risk and not offered chemotherapy may develop recurrent disease, because of an underlying and unnoticed higher risk that, if recognized, would support the consideration of chemotherapy. The use of histological grade and Ki67 expression provides prognostic information and support for clinical risk classification. However, both parameters are subjected to inter- and intra-laboratory variations, challenging the establishment of robust thresholds for adjuvant systemic treatment decisions.^7^, ^8^, ^9^, ^10^, ^11^

To better stratify patients and improve decision making for systemic therapy, several molecular gene expression classifiers have been established.^12^, ^13^, ^14^, ^15^, ^16^, ^17^ These assays provide superior prognostic information to conventional histopathological assessments.^16^^,^^18^, ^19^, ^20^, ^21^, ^22^, ^23^, ^24^ Two large prospective trials using OncotypeDx and MammaPrint (TAILORx^25^ and MINDACT^16^^,^^26^) showed very low rates of recurrence with adjuvant endocrine treatment alone (ET) in patients with HR+/HER2− pN0 EBC and favourable gene expression pattern. Similar findings were also reported in prospective retrospective studies of other gene expression assays such as Prosigna, Breast Cancer Index and EndoPredict.^23^^,^^27^, ^28^, ^29^, ^30^ Thus, to identify patients with a low risk of recurrence, molecular gene expression classifiers have recently been included in clinical guidelines on adjuvant systemic treatment decisions for this patient population.^31^, ^32^, ^33^, ^34^, ^35^, ^36^ However, the optimal routine use of molecular gene expression classifiers throughout the clinical risk spectrum is still, in part, subjected to uncertainty.

Despite the growing evidence for use of gene expression profiles as a component of the risk assessment, the access to molecular profiling varies around the world. This is due to inconsistencies in acceptable cost of care delivery between countries and differences in the extent of public health services, as well as delays in making new methodology and treatment available at an affordable cost.^37^ Thus, histological grade and Ki67 are still used to guide adjuvant treatment decisions in HR+/HER2− pN0 EBC.^36^ Although real-world data exist for several molecular gene expression classifiers,^38^, ^39^, ^40^, ^41^, ^42^, ^43^, ^44^ data on the use of the Prosigna test are limited.^41^^,^^42^ There is need for larger prospective decision impact studies evaluating how the Prosigna test influences adjuvant treatment decisions as well as the cost-effectiveness of the test.

EMIT^EBC^-1 (Establishment of Molecular Profiling for Individual Treatment decision in Early Breast Cancer; NCT03904173) is a national, prospective, observational multicentre single-arm trial designed to assess the value of Prosigna as a routine diagnostic tool, examining its impact on adjuvant treatment decisions (versus standard histopathology including Ki67), clinical outcomes, long-term side-effects and cost-effectiveness. A complete description is presented in the protocol (available alongside this article). The first part of the study aims to assess how the Prosigna test in addition to standard histopathology affects the risk classification and adjuvant treatment decision in an unselected HR+/HER2− axillary node-negative EBC study population.

## Patients and methods

### Study population

**Figure 1:**
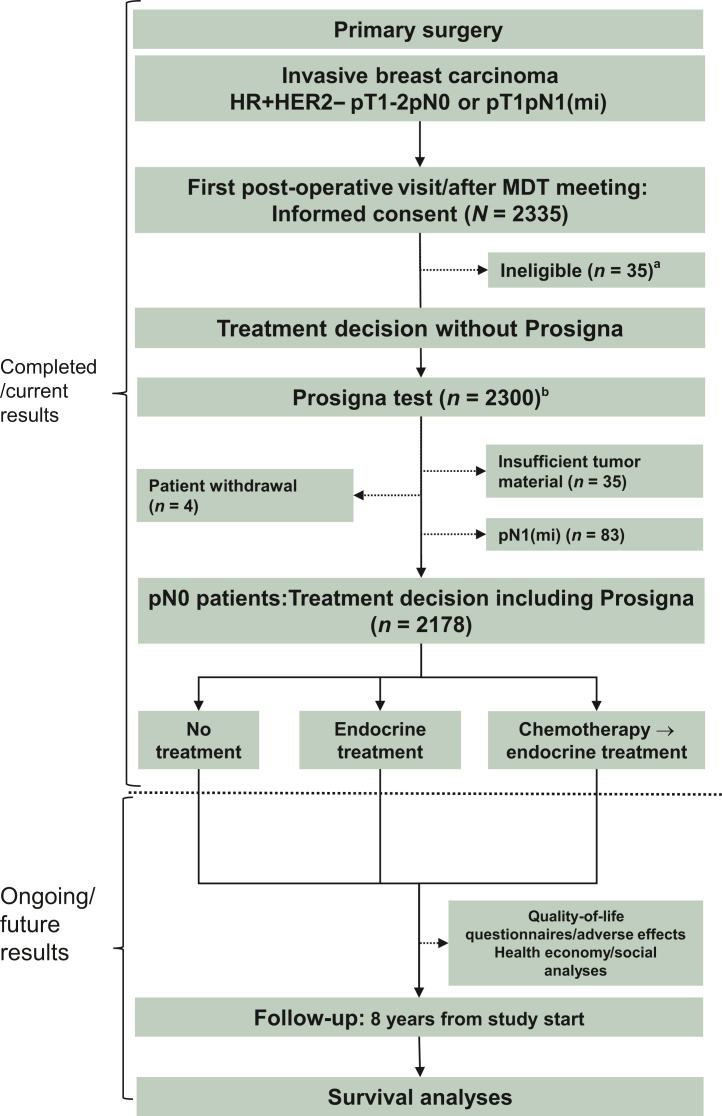
**Schematicoverview of the study**. ^a^Ineligible: Previous or concurrent cancer diagnosis (*n* = 11), distant metastases (*n* = 1), HER2+ (*n* = 2), pT2 N1(mi) (*n* = 6), DCIS only (*n* = 1), pN1 (*n* = 3), pNx due to other diseases (*n* = 3), patient not able to comply with protocol (*n* = 1) and screening failure, reason not specified (*n* = 7). ^b^Prosigna test not applicable: Patient consent withdrawn before test (*n* = 6), insufficient tumour material for RNA extraction or low tumour cell count (*n* = 28), other reasons not specified (*n* = 1). Study start: 01 October 2018. DCIS, ductal carcinoma *in situ*; HER2, human epidermal growth factor receptor 2; HR, hormone receptor; MDT, multidisciplinary team.

During the period 2018-2022, early breast cancer patients with HR+/HER2− pT1-T2 pN0 disease from 17 Norwegian hospitals (including all Norwegian health regions) were recruited in the prospective observational adjuvant EMIT-1 study (Figure 1). As of October 2020, the protocol was revised to allow for the enrolment of patients with micrometastasis to axillary lymph nodes [pN1(mi)] if pT1. Participating patients signed informed consents before study-specific analyses and all procedures described were carried out in accordance with The Code of Ethics of the World Medical Association (Declaration of Helsinki)^45^ for experiments involving humans. An *a priori* sample size calculation was carried out for the primary endpoint (distant recurrence-free interval 8 years after study start) with the aim to include 2150 lymph node-negative patients. A total of 2335 patients were enrolled, and 35 were excluded because they did not fulfil the inclusion criteria. In the current assessments, patients with pN1(mi) (*n* = 83), with insufficient tumour material for RNA extraction to carry out the Prosigna test analysis (*n* = 35) or withdrawal before the final treatment decision (*n* = 4) were not included. Thus, 2178 patients were included in the analyses (Figure 1).

### Biomarker analyses

Histopathological tumour characterization and standard biomarker analyses were carried out at the local pathology laboratory according to the World Health Organization and Norwegian guidelines^46^^,^^47^ and the results were obtained from the Cancer Registry of Norway. Estrogen receptor (ER), progesterone receptor (PR) and HER2 were assessed using immunohistochemistry (IHC) according to national procedures, including *in situ* hybridization for HER2 (two-step protocol). ER was defined as positive if ≥1% and PR positive if ≥10% of the tumour cell nuclei stained positive. If HER2 IHC scored 2+, the analysis was supplemented by *in situ* hybridization (FISH/CISH/SISH) and defined negative when the *HER2/CEP17* ratio was <2 and average *HER2* copy number was <6 per tumour cell, or the *HER2/CEP17* ratio was >2 and average *HER2* copy number was <4 per cell, according to the American Society of Clinical Oncology (ASCO)-College of American Pathologists guidelines (2018). Ki67 proliferation index was reported as the percentage of positively stained tumour cell nuclei in the hot spot (HS) according to national guidelines (www.nbcg.no). Ki67 was analysed as continuous and categorical (≥/< median; <10%, 10%-35%, >35%) variable. The cut-offs 10% and 35% were based on the Ki67 thresholds used in the St Gallen consensus recommendations (2021) and the ASCO guidelines (5%/30%).^33^^,^^36^ The thresholds were adjusted to account for the difference between Ki67 HS and an average method with a minimum of 5 units, in line with results from our previous study (Supplementary Figure S1, available at https://doi.org/10.1016/j.esmoop.2024.103475). Due to inter-laboratory variations in absolute Ki67 scores as well as potential inter-hospital variations in the clinical interpretation of high and low Ki67 values based on the hospitals’ median Ki67 values, Ki67 scores were normalized to the local laboratory median Ki67 score and categorized as <0.5× median (low), 0.5-1.5× median (intermediate) and >1.5× median (high) for comparison between hospitals.

### Intrinsic subtype and Risk of Recurrence score

A representative tumour block was selected by the local pathologist, preferably the same block used for Ki67 testing, and sent to the Central Laboratory for Prosigna testing (one national central laboratory until regional laboratories were certified for the Prosigna procedure and the tests were carried out in their respective labs; see Supplementary Information, available at https://doi.org/10.1016/j.esmoop.2024.103475, for details). RNA was extracted using the Roche FFPET RNA Isolation kit (Roche, Pleasanton, CA), catalogue number 025 from macro-dissected, formalin-fixed paraffin-embedded tissue sections from breast cancer tumour tissue. Expression of the Prediction Analysis of Microarray using the 50-gene classifier (PAM50) genes was analysed on the nCounter Analysis System (NanoString, Seattle, WA) using the Prosigna® Breast Cancer Prognostic Gene Signature Assay (Veracyte, South San Francisco, CA), the output of which generates the tumour molecular subtype, the Risk of Recurrence (ROR) score and risk category. Patient samples that did not pass quality threshold for the Prosigna analysis were re-analysed. Tumours were categorized as low, intermediate or high risk of recurrence based on ROR scores ≤40, 41-60 and >60, respectively.

### Clinical treatment decisions

As support for the selection of adjuvant treatment, clinicians use consensus recommendations and guidelines based on prognostic and predictive information.^32^^,^^33^^,^^36^^,^^47^ This may also include ‘calculators’ such as the previously used web-based algorithm Adjuvant! Online.^48^ In Norway, patients with HR+/HER2− pT1-pT2 pN0 disease have traditionally been assigned to treatment/clinical risk categories based on menopausal- and T-status, ER and Ki67 expression as well as histological grade, as presented in Supplementary Table S1, available at https://doi.org/10.1016/j.esmoop.2024.103475. When the study was initiated, the national recommendation on how to interpret the Ki67 results for treatment decisions was based on the St Gallen consensus recommendations from 2015 and 2017.^49^^,^^50^ Patients with especially low clinical risk (IHC luminal A-like pT1a-b or pT1c histological grade 1) typically do not receive any systemic adjuvant treatment. The clinically higher-risk patients include those with IHC luminal B-like features (if not pT1a-b) or premenopausal patients with IHC luminal A-like pT2 tumours. These patients are recommended chemotherapy followed by endocrine treatment (CT + ET). The remaining patients (intermediate-risk group) are assigned to ET. In line with the study protocol, the local physicians (primarily the multidisciplinary team) recorded their recommended, Norwegian guideline-based adjuvant treatment decision, before (pre-Prosigna) and after (post-Prosigna) the Prosigna test result was available (Figure 1, Supplementary Table S1, available at https://doi.org/10.1016/j.esmoop.2024.103475, and www.nbcg.no). The treatment decision algorithm including the Prosigna test results integrates both the ROR score category and intrinsic PAM50 subtype classification for treatment recommendation (Supplementary Table S1, available at https://doi.org/10.1016/j.esmoop.2024.103475). The pre-Prosigna treatment decision was blinded for the Prosigna test result. In addition, the local physicians recorded their pre-Prosigna treatment decision when patient-related factors (including patient preferences, comorbidity, age, specific tumour characteristics or overall assessment) were taken into consideration. Eventually, the final treatment decision (actual treatment started) was registered.

The parameters used for risk assessment converted into treatment guidelines are not categorical, but inform recurrence risk as a continuum. The physicians’ confidence with the treatment decision may therefore vary. Hence, following the pre-Prosigna treatment decisions, the local physicians or multidisciplinary team (whoever made the decision) also recorded if they were confident (yes/no) with the decision.

To compare treatment decisions across hospitals, sites with >50 included patients were chosen (see Supplementary Information, available at https://doi.org/10.1016/j.esmoop.2024.103475). Recruitment of patients with pT1a-b tumours varied between hospitals and there were few chemotherapy candidates among these. Thus, patients with pT1c-T2 tumours were selected for this comparison. Based on histopathological grade and Ki67 level, patients were grouped (pre-Prosigna test) as ‘No-chemo candidates’ (G2 and Ki67 <0.5× median or G1 and Ki67 <1× median), ‘Uncertain chemo candidates’ (G2 and Ki67 0.5-1.5× median) and ‘Chemo candidates’ (G2 and high Ki67 >1.5× median or G3 and Ki67 >1× median).

### Statistics

Descriptive statistics were used for patients and tumour characteristics and presented as frequencies and proportions for categorical variables and as median and range for continuous variables. To compare groups, Pearson’s ꭕ^2^ and Kruskal–Wallis tests were carried out. Correlations were assessed by Pearson’s *r* and *R*^2^. Graphically, scatterplot, violin plot and Sankey plot (Sankeymatic.com) were used for illustration. All *P* values were two-tailed and *P* < 0.05 was regarded as statistically significant. There were no missing data in the current dataset.

## Results

A total of 2178 pN0 patients had conclusive primary tumour Prosigna test results. Clinical and demographic characteristics are shown in Table 1. The median age at diagnosis was 60 years. Characteristics of the total study population [including patients with pN1(mi)] and for patients without conclusive Prosigna test results or no post-test treatment decision are presented in Supplementary Table S2, available at https://doi.org/10.1016/j.esmoop.2024.103475. Corresponding epidemiological data from the Cancer Registry of Norway are shown in Supplementary Table S3, available at https://doi.org/10.1016/j.esmoop.2024.103475.

**Table 1:** Characteristics of patients in the primary analysis (*n* = 2178)

Characteristics	*n* (%)
Sex	
Female	2166 (99)
Male	12 (0.6)
Age (years)	
Median (range)	60 (19-89)
Categories	
<40	72 (3.3)
40-50	368 (17)^a^
51-60	724 (33)^b^
61-70	759 (35)
>70	255 (12)
Menopausal status^c^	
Premenopausal^d^	539 (25)
Postmenopausal	1565 (72)
Unknown	49 (2.2)
Male	12 (0.6)
Missing	13 (0.6)
T-category	
pT1a	63 (2.9)
pT1b	466 (21)
pT1c	1129 (52)
pT2	520 (24)
N-category	
pN0	2115 (97)
pN0 (i+)	63 (2.9)
Ki67	
Median (range)	19 (1-92)
Histological grade	
G1	518 (24)
G2	1290 (59)
G3	370 (17)
ER	
Positive (1-<10%)	9 (0.4)
Positive (10-<50%**)**	36 (1.7)
Positive ≥50%	2118 (97)
Positive, unspecified	15 (0.7)
PR	
Negative <10%	349 (16)
Positive (10%-<50%)	343 (16)
Positive ≥50%	1482 (68)
Positive, unspecified	4 (0.2)
PAM50 subtype	
Luminal A	1352 (62)
Luminal B	788 (36)
HER2 enriched	18 (0.8)
Basal-like	20 (0.9)
ROR category	
0-40	1059 (49)
41-60	686 (31)
>60	433 (20)
ROR score	
Median (range)	41 (0-94)

ER, estrogen receptor; PAM50, Prediction Analysis of Microarray using the 50-gene classifier; PR, progesterone receptor; ROR, Risk of Recurrence.

a 43 patients (12%) postmenopausal.

b 527 patients (73%) postmenopausal.

c Menopausal status at trial entry.

d Premenopausal includes patients with regular menstruation (*n* = 300), menstrual irregularities (*n* = 75) and hormonal intrauterine device (*n* = 164).

The majority of tumours were classified by Prosigna as intrinsic luminal A subtype (62%), 36% were luminal B, and 0.8% and 0.9% were HER2-enriched or basal-like subtypes, respectively. The ROR scores were ≤40 in 49%, 41-60 in 31% and >60 in 20% of tumours (ROR score grouped by subtype, see Supplementary Figure S2, available at https://doi.org/10.1016/j.esmoop.2024.103475). For patients younger than 50 years, 61% had luminal A and 37% had luminal B tumours; the ROR scores were ≤40 in 47%, 41-60 in 32% and >60 in 21% of tumours. The distribution of intrinsic subtypes and ROR scores per age category is displayed in Supplementary Table S4, available at https://doi.org/10.1016/j.esmoop.2024.103475.

### Clinical treatment decisions

Based on national guidelines for risk profile assessment without Prosigna, the local physicians’ pre-Prosigna treatment decisions were no systemic treatment (NT) in 27% of patients, ET in 38% and CT + ET in 35% (Table 2). The assignment to the treatment groups changed minimally (≤1%) in favour of endocrine treatment (Supplementary Table S5, available at https://doi.org/10.1016/j.esmoop.2024.103475) when patient-related factors were included. Using the modified Adjuvant! Online categorization as carried out in the MINDACT trial, 31% of patients were classified as clinical high risk and 69% as clinical low risk (Supplementary Table S6, available at https://doi.org/10.1016/j.esmoop.2024.103475). Of the patients assigned to chemotherapy pre-Prosigna, 62% were categorized as clinical high risk, whereas 86% of the patients assigned to no chemotherapy were clinical low risk (with modified Adjuvant! Online).

**Table 2:** Treatment decisions^a^ pre- and post-Prosigna test (*n* = 2178)

Treatment decision	Pre-Prosigna *n* (%^b^)	Post-Prosigna *n* (%^b^)
No systemic treatment (NT)		
All	596 (27)	555 (25)
≤50 years	85 (19)	80 (18)
>50 years	511 (29)	475 (27)
Endocrine treatment alone (ET)		
All	818 (38)	1102 (51)
≤50 years	140 (32)	220 (50)
>50 years	678 (39)	882 (51)
Chemotherapy + endocrine treatment (CT + ET)		
All	764 (35)	521 (24)
≤50 years	215 (49)	140 (32)
>50 years	549 (32)	381 (22)

ER, estrogen receptor.

a Without patient-related factors.

b % values calculated in the pN0 study population for all (*n* = 2178) patients, for patients ≤50 (*n* = 440) or >50 (*n* = 1738) years in the respective treatment decision groups. Excluding patients with ER expression <10% (*n* = 9) did not change the distribution of treatment decisions.

The decision to offer chemotherapy was more frequent in patients 50 years or younger (Table 2; with patient-related factors shown in Supplementary Table S7, available at https://doi.org/10.1016/j.esmoop.2024.103475). Furthermore, the physicians were uncertain about the NT decision in 12%, the ET decision in 27% and the CT + ET decision in 36% of the patients. They were also less confident in recommending endocrine therapy alone to younger patients than to older patients, and more confident in assigning them to chemotherapy (Supplementary Table S8, available at https://doi.org/10.1016/j.esmoop.2024.103475).

Guideline-based treatment decisions including the Prosigna test resulted in 25% of patients being assigned to NT, 51% to ET and 24% to CT + ET (Table 2). After also considering patient-related factors, the final treatment decision was 26%, 52% and 22%, respectively (Supplementary Table S9, available at https://doi.org/10.1016/j.esmoop.2024.103475). Reasons to deviate from national guidelines were patient preferences (*n* = 45), comorbidity (*n* = 11), overall assessment (*n* = 31) and other reasons not specified (*n* = 4).

### Treatment decision alteration

**Figure 2:**
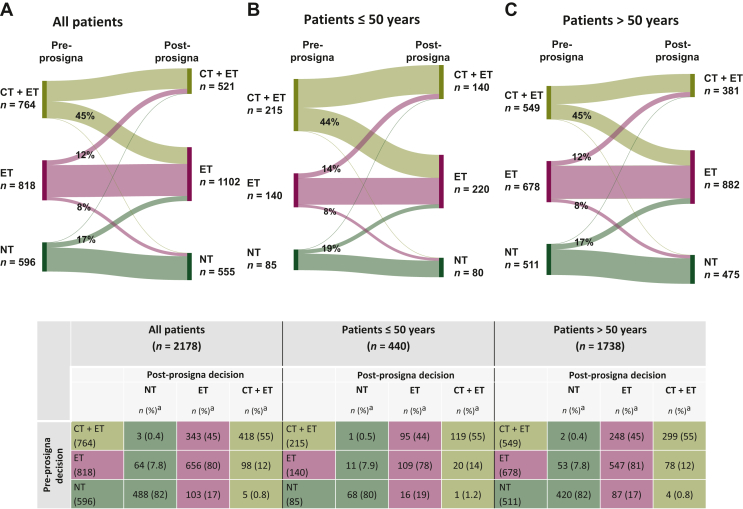
**Treatment decision alterations**. Treatment decision alteration for (A) pN0 total study population with conclusive Prosigna test result, (B) patients aged ≤50 years and (C) patients >50 years of age. Pre-test: Treatment decision before knowledge of the Prosigna test result. Post-test: Treatment decision after the Prosigna test result was declared. CT, chemotherapy; ET, endocrine therapy; NT, no systemic treatment. ^a^% of Pre-Prosigna decision.

Adjuvant treatment decisions changed in 28% (*n* = 616) of patients, including 21% (*n* = 449) change in chemotherapy use. The treatment decision alterations (pre- versus post-Prosigna test) are shown in Figure 2 (per site; Supplementary Figure S3, available at https://doi.org/10.1016/j.esmoop.2024.103475).

In the group assigned to CT + ET pre-Prosigna, 45% of patients were de-escalated to ET after disclosure of the Prosigna test result (Figure 2). This was independent of age. However, including patient-related factors, younger-age patients (≤50 years) remained on chemotherapy treatment decision more often than patients aged >50 years (59% versus 51%) (Supplementary Table S10, available at https://doi.org/10.1016/j.esmoop.2024.103475). Of patients assigned to ET alone pre-Prosigna, 12% were escalated to CT + ET and 8% de-escalated to NT. For patients assigned to NT pre-Prosigna, 18% were escalated to either ET (*n* = 103) or CT + ET (*n* = 5) post-Prosigna.

The treatment escalations were more frequent among patient aged ≤50 years, but the differences were small (Figure 2B, C).

According to clinical risk categorization as carried out in the MINDACT trial (modified Adjuvant! Online), the post-Prosigna treatment decision was no CT for 44% of the clinical high-risk patients and CT + ET for 9.3% of the clinical low-risk patients (Supplementary Table S6, available at https://doi.org/10.1016/j.esmoop.2024.103475).

### Treatment decisions across hospitals

**Figure 3:**
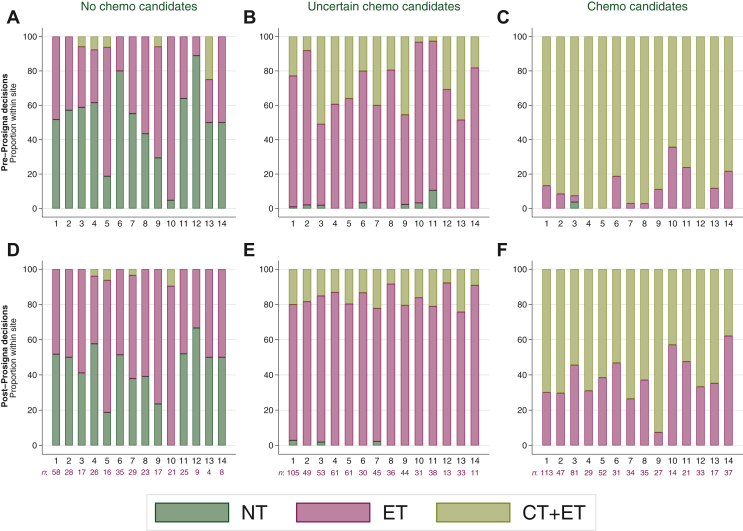
**Treatment decision across hospitals (site 1-14, with more than 50 patients included) for patients with pT1c-pT2 pN0.** No-chemo candidates: G2 and Ki67< 0.5× local laboratory median score or G1 and Ki67 <1× local laboratory median score. Uncertain chemo candidates: G2 and Ki67 0.5-1.5× local laboratory median score. Chemo candidates: G2 and Ki67 >1.5× local laboratory median score or G3 and Ki67 >1× local laboratory median score. Treatment decision without Prosigna test (A-C). Treatment decision with Prosigna test (D-F). CT, Chemotherapy; ET, endocrine therapy; NT, no systemic treatment.

Focusing on the pT1c-pT2 patients where chemotherapy typically is considered, the patients were divided into groups according to the likelihood of being chemotherapy candidates (‘no-chemo/uncertain/chemo candidates’, see Materials and Methods). The distribution of treatment decisions pre-Prosigna test varied widely across hospitals (Figure 3). In particular, for patients within the ‘uncertain chemo candidate’ group, the use of chemotherapy ranged from 3% to 51% (Pearson’s ꭕ^2^ 91.7, *P* < 0.001, Figure 3B).

After the Prosigna test results were revealed, the frequency of the decision ET increased in all patient groups. The treatment decisions across hospitals became more evenly distributed, in particular for the ‘uncertain chemo candidates’ (i.e. the group of patients with G2 and intermediate Ki67). For these patients the variability in chemotherapy use was reduced to 8%-24% (Pearson’s ꭕ^2^ 18.7*, P* = 0.85, Figure 3E). In the ‘no-chemo’ and ‘chemo candidate’-groups, the treatment decision showed larger variation across hospitals. For the ‘chemo candidate’ subgroup, patients with ROR score >60 ranged from 24% to 78% across the hospitals. Similarly, for the ‘no-chemo candidate’ group, patients with ROR score ≤40 ranged from 52% to 100% (Supplementary Table S11, available at https://doi.org/10.1016/j.esmoop.2024.103475).

### Clinicopathological and molecular correlates

Among all patients, in the group assigned to NT pre-Prosigna the majority of tumours (90%) were intrinsic luminal A subtypes with low ROR score (82%). However, as many as 9.4% of these were intrinsic luminal B subtypes and 17% had intermediate ROR score (Table 3). Conversely, in the group assigned to CT + ET pre-Prosigna, one-third of tumours were intrinsic luminal A subtype and 56% had low or intermediate ROR score (18% and 38%, respectively).

**Table 3:** Clinical profiles and molecular subtypes (*n* = 2178)

	Pre-Prosigna decision	Pre-Prosigna decision	Pre-Prosigna decision
	No treatment, *n* (%)	Endocrine alone, *n* (%)	Chemo + endocrine, *n* (%)
PAM50 subtype			
Luminal A, *n***= 1352**	539 (90)	571 (70)	242 (32)
Luminal B, *n* = 788	56 (9.4)	244 (30)	488 (64)
HER2 enriched, *n* = 18	1 (0.2)	2 (0.2)	15 (2.0)
Basal-like, *n* = 20	0	1 (0.1)	19 (2.5)
ROR score			
Median (range)	27 (0-84)	39 (0-89)	58 (10-94)
0-40, *n* = 1059	488 (82)	434 (53)	137 (18)
41-60, *n* = 686	99 (17)	296 (36)	291 (38)
>60, *n* = 433	9 (1.5)	88 (11)	336 (44)

PAM50, Prediction Analysis of Microarray using the 50-gene classifier; ROR, Risk of Recurrence.

**Figure 4:**
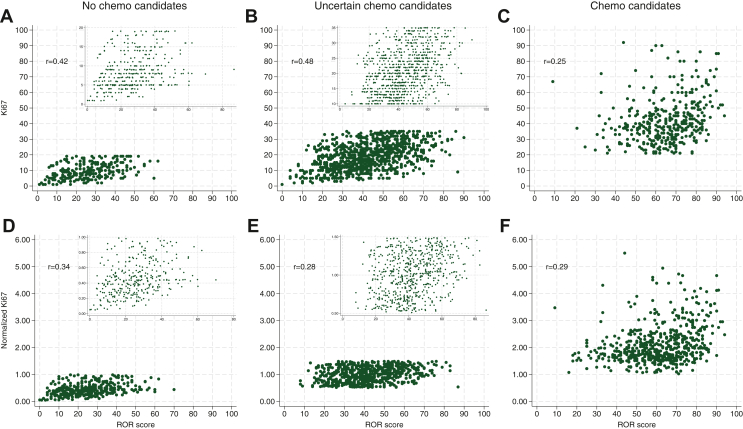
**Ki67-Risk Of Recurrence** (ROR) **scatterplot of pT1c-pT2 tumours with Ki67 analysed as continuous variable and presented as absolute score value (‘Absolute Ki67’) or normalized to the local laboratory median Ki67 score (‘Normalized Ki67’).** (A) G2 and Ki67 <10% or G1 and Ki67 <20%, (B) G2 and Ki67 10%-35%, (C) G2 and Ki67 >35% or G3 and Ki67 >20%, (D) G2 and Ki67 <0.5× local laboratory median score or G1 and Ki67 <1× local laboratory median score, (E) G2 and normalized Ki67 0.5-1.5× local laboratory median score, and (F) G2 and normalized Ki67 >1.5× local laboratory median score or G3 and normalized Ki67 >1× local laboratory median score. In (A-B and D-E), the parts of the figures containing data points are enlarged in the upper right corner.

The linear correlation between Ki67 and ROR score was 0.66 (Pearson’s *r* 0.66, *P* < 0.001, *n* = 2178*,* Supplementary Figure S4, available at https://doi.org/10.1016/j.esmoop.2024.103475) and the variation across hospitals was large (*r* = 0.41-0.82/*R*^2^ = 0.17-0.67). In the clinical subgroups of pT1c-pT2 patients separated by a likelihood of receiving chemotherapy (‘no-chemo/uncertain-/chemo candidates’), the correlation between Ki67 expression and ROR score within the subgroups was poor (*r* = 0.25-0.39), both if the absolute Ki67 and normalized Ki67 values were used (Figure 4).

Similarly, we observed a positive association between histological grade and ROR categories (Pearson’s ꭕ^2^ *P* < 0.001), as well as with ROR score as a continuous variable (Kruskal–Wallis *P* < 0.001, Supplementary Figure S5A, available at https://doi.org/10.1016/j.esmoop.2024.103475). However, the ROR score ranges were wide (for G1 0-79, G2 0-90, G3 16-94). The same was observed for the modified Adjuvant! Online clinical risk categories (clinical low risk 0-89, clinical high risk 8-94) (Supplementary Figure S5B, available at https://doi.org/10.1016/j.esmoop.2024.103475).

## Discussion

In this large prospective, observational study, the Prosigna gene expression test identified patients considered to be at both lower and higher risk than what was judged by clinical risk stratification using conventional histopathology. The Prosigna test result changed adjuvant treatment decisions in all EBC clinical risk groups, markedly decreased the CT use for patients categorized as higher clinical risk pre-Prosigna and reduced treatment decision discrepancies between hospitals. Overall, the treatment decision was altered for 28% of the patients, including 21% change in chemotherapy decisions. This treatment alteration rate is slightly higher than the rate seen in older, small decision impact studies evaluating Prosigna clinical utility.^51^^,^^52^

Our results suggest that 45% of patients assigned to chemotherapy by routine classification can forgo this treatment when the Prosigna test is incorporated in the decision algorithm. This is in line with results from other real-life studies.^41^^,^^44^ Accordingly, their tumours presented with low or intermediate ROR score, thus overall low risk of developing future overt metastatic disease, at least if postmenopausal.^22^^,^^23^^,^^26^ Safely omitting chemotherapy will reduce overtreatment and concomitant treatment-related side-effects experienced by a majority of the patients, although to a variable degree.^53^ Thus, undesirable toxicities, economic disadvantages for the individual patient and societal costs may be reduced.

The use of Prosigna testing also led to more consistent treatment decisions across hospitals, which is important to ensure a standardized basis for treatment nationwide. To our knowledge, this is the largest and most comprehensive study comparing ROR score and Ki67 expression, with the potential to unravel the usefulness and variability of Ki67 both at the patient level and across hospitals. When treatment decisions were based on conventional pathology criteria alone, a considerable variation between hospitals was observed, e.g. ranging from 3% to 51% in chemotherapy use for patients with pT1c-pT2 G2 and intermediate Ki67 expression (Figure 3C). Most EBC HR+/HER2− tumours fall into this group.^54^ This emphasizes that current routine diagnostic analyses are unable to provide reliable information to make clear treatment recommendations on chemotherapy use for a large number of patients. Furthermore, the results underpin the clinical variability in the interpretation of histopathological markers. The International Ki67 in Breast Cancer Working Group^7^ does not support using Ki67 values between 5% and 30% (average scores) for clinical decision making on chemotherapy. The Prosigna test, on the other hand, provides more prognostic information and has shown superior inter-laboratory reproducibility suiting a decentralized diagnostic strategy.^20^^,^^55^ In our study, the more similar treatment decisions after incorporating the Prosigna test results were most distinct among patients classified as ‘uncertain’ chemotherapy candidates (pT1c-pT2, intermediate Ki67 (HS) and G2). For the ‘no-chemo’ and ‘chemo’ candidate groups, larger variability persisted also post-Prosigna. Disparities in patient inclusion (see Materials and Methods) as well as differences in tumour characteristics (i.e. ROR distribution width) are probably contributing to this variability. Overall, the improved agreement in treatment decisions in our study is in line with what was reported in a substudy within the MINDACT cohort.^56^

The imbalanced access to molecular profiling around the world accentuates the question of Ki67 as a surrogate marker for ROR score. In accordance with a recent (but smaller) real-world population study,^42^ we observed poor correlation between Ki67 and ROR score among tumours with intermediate Ki67 (i.e. uncertain chemotherapy candidates), but also in the other clinical risk groups. Thus, the results in the ‘uncertain chemo group’ strongly support the Ki67 working group recommendation to avoid use of Ki67 in the intermediate range for adjuvant treatment decisions. Similarly, the ROR score ranges in the separate histological-grade categories were wide. The presence of histological grade 3 is established as a poor prognostic factor, but the large histological grade 2 group biologically seems to constitute a mixture of biological low- and high-grade tumours.^57^ Further sub-classification of especially grade 2 breast tumours is therefore of importance.^58^ Altogether, in the present study, neither histological grade nor Ki67 were adequate as parameters to select tumours for molecular profiling.

Although the use of molecular gene expression classifiers to support adjuvant treatment decisions is well documented, their usefulness for omission of chemotherapy in intermediate-/higher-risk premenopausal women is not settled. In the current study, patients ≤50 years of age were more frequently assigned to chemotherapy, both pre-Prosigna and post-Prosigna, compared to older patients. This was further augmented when patient-related factors were considered, and if premenopausal patients with regular menstruation were included (data not shown). Updated results with explorative analyses from both TAILORx^59^ and MINDACT^26^ trials may indicate that the comparable (and excellent) distant metastasis-free survival with ET versus CT + ET appears to be age dependent with potential benefit of adding chemotherapy for patients ≤50 years of age. However, the use of ovarian function suppression was limited in both trials (13% and 21%, respectively) and probably influenced the results. Nevertheless, due to the results from these studies, our guidelines shifted towards increased use of chemotherapy in higher-risk premenopausal patients in 2021 (Supplementary Table S1, available at https://doi.org/10.1016/j.esmoop.2024.103475). In our study, the majority of the ≤50 years of age subgroup consisted of lower-risk patients, and 51 out of the 95 patients who were de-escalated to no chemotherapy were clinical low-risk patients when using the modified Adjuvant! Online risk classification as carried out in the MINDACT study. These patients would not have been eligible for the randomization to chemotherapy in that study. Furthermore, 86% of the clinical high-risk (as in MINDACT) younger patients de-escalated to no chemotherapy were luminal A by Prosigna (data not shown). However, poor prognostic features may be enriched in younger patients with HR+ breast cancer.^60^ In our study, patients ≤40 years of age were de-escalated to endocrine therapy alone much less frequently than the other age groups (only 10 patients out of 72). Resolving the question of age-related differences in the benefit of adjuvant chemotherapy requires additional, randomized studies including ovarian function suppression for all. The ongoing OPTIMA study addresses this issue for lymph node-positive, HR+/HER2− patients, including both pre- and postmenopausal women (ISRCTN42400492).

Endocrine treatment is recommended to all or most HR+/HER2− EBC patients in many countries and guidelines. Although chemotherapy toxicity has received most attention, side-effects from endocrine treatment may also negatively affect different aspects of life quality and lead to non-adherence with subsequent potential higher recurrence risk.^61^ Thus, identifying patients with limited absolute benefits from endocrine treatment, despite expected similar relative benefits, will probably improve shared decision making. In the present study, 8% of patients assigned to ET using standard histopathology criteria were transferred to no adjuvant systemic treatment when the Prosigna test was included in the decision making. In addition, 82% of the patients recommended no adjuvant systemic treatment pre-Prosigna were confirmed to belong to an especially low genomic risk profile. Although none of the gene expression profiles so far have been validated for de-escalation of endocrine therapy, our previous results showed that the patient population with low ROR score will most likely have an excellent prognosis receiving no adjuvant treatment (i.e. breast cancer death 4.0% after 17 years of follow-up without systemic treatment).^24^ This is consistent with a subpopulation of smaller tumours in the STO-3 trial^62^ and with the ultralow-risk group developed from the 70-gene signature (MammaPrint) with an exceptionally good prognosis after 20 years of follow-up.^63^^,^^64^ De-escalation of/shortened endocrine therapy guided by Prosigna will also be evaluated in the LA LEAST study (NCT03917082), which will provide a useful comparison for optimizing endocrine therapy strategies.

The Prosigna test also identified patients considered to be at higher risk than what was implied by the risk stratification pre-Prosigna (using standard histopathological criteria). A substantial proportion of patients within the low-risk group (assigned to NT pre-Prosigna) and intermediate-risk group (assigned to ET pre-Prosigna), were recommended ET and CT + ET post-Prosigna (18% and 12%, respectively). In small tumours (pT1a-b N0) molecular profiling has an uncertain role and is generally not advised because the test result would not influence the use of chemotherapy.^50^^,^^65^ In accordance, our results show that <1% of patients within the no-treatment group pre-Prosigna were recommended chemotherapy post-Prosigna. However, if no adjuvant systemic treatment is considered as an option, the Prosigna test identifies those higher-risk patients who definitely should be offered endocrine treatment.^24^^,^^66^ Furthermore, in the ScanB study the Prosigna-based guideline for treatment decision in our study (Supplementary Table S1, available at https://doi.org/10.1016/j.esmoop.2024.103475) was retrospectively applied to patients who had received adjuvant endocrine treatment. The subgroup with a PAM50/ROR score corresponding to chemotherapy advice had poor survival with ET (hazard ratio 4.9, 95% confidence interval 1.94-8.62).^66^ Similarly, patients receiving ET with high ROR score in the DBCG study had a markedly increased cumulative incidence of distant recurrence compared to patients with low ROR score.^23^ Without proven predictive tools for chemotherapy sensitivity and acknowledging the relative average effect of adjuvant chemotherapy, a markedly higher recurrence risk supports the use of chemotherapy. This implies a potential for using the Prosigna test also to avoid under-treatment. The MINDACT study showed a small numerical improvement in distant metastasis-free survival for the chemotherapy arm among clinical low-/genomic high-risk patients and a 2.9% increase in 8-year distant metastasis-free interval [hazard ratio 0.61 (95% CI 0.34-1.07)], but the study was not powered for a conclusive answer to this escalation question.^26^

Despite studies and recommendations supporting the clinical use of molecular gene expression classifiers for HR+/HER2− breast cancer, the access to these expensive tests around the world are imbalanced. Cost-effective analyses are critical to clarify the implications for health care and societal costs within the different risk groups, giving support for knowledge-based decisions on reimbursement and budgeting. Several studies have been carried out for other gene expression classifiers, including routine practice,^67^ but to our knowledge there are few real-world cost-effectiveness studies on the Prosigna test. This is one of the objectives in the EMIT^EBC^-1 study and analyses are ongoing.

Due to its observational study design, this study has potential limitations, including risk of introducing selection bias. Although principally representative for HR+/HER2− lymph node-negative population in Norway with respect to age distribution, tumour size and grade (Cancer Registry of Norway, Supplementary Table S3, available at https://doi.org/10.1016/j.esmoop.2024.103475), the study cohort included some imbalances across hospitals, particularly for small tumours and in the older age group (>70 years). Furthermore, the treatment decisions could be affected by comorbidity, age and performance status. However, the study registered treatment decisions based on guidelines alone compared to decisions including patient-related factors, with minimal differences. As a single-armed trial, the study cannot demonstrate the prognostic treatment intervention effect of using the Prosigna test compared to standard histopathological risk factors. Nevertheless, the study is designed as a decision impact study, including the opportunity to evaluate the use of new diagnostic practice in a real-world setting, also comprising elderly patients and those with comorbidity, as well as identifying gaps in the care between hospitals.

### Conclusion

In this real-world study, the Prosigna test result changed adjuvant treatment decisions in all EBC clinical risk groups, markedly decreased the CT use for patients categorized as higher clinical risk pre-Prosigna and reduced treatment decision discrepancies between hospitals. Although clear evidence for the benefit of treatment changes in low-risk groups based on the Prosigna result is lacking, the study will provide further insight when follow-up data are available. Including the Prosigna test in the decision algorithm can improve the prognostic classification in pT1-pT2 N0 breast cancer, allowing more precise and uniform identification of future recurrence risk and improved basis for adjuvant treatment decisions. Ultimately, our study will provide information on the cost-effectiveness and societal impact of using Prosigna as part of adjuvant treatment decision, including collected health care resource use, quality-of-life measurements, assessment of work ability and side-effects from treatment, as well as survival.
