# Supplementary material for: Impact of Prosigna test on adjuvant treatment decision in lymph node-negative early breast cancer—a prospective national multicentre study (EMIT-1)
Source: ESMO Open. 2024 Jun 4;9(6):103475. doi: 10.1016/j.esmoop.2024.103475 (PMC11190479; doi:10.1016/j.esmoop.2024.103475)
Supplement: Supplementary Information [file mmc1.pdf]

Supplementary Information

Norwegian Laboratories certified to perform the Prosigna procedure:

Oslo University Hospital: June 2018

University of North Norway: May 2019

Haukeland University Hospital: April 2020

St Olav Hospital: April 2020

Stavanger University Hospital: January 2022

Additional information concerning selection of tissue for Prosigna-testing

Preferably the block containing the largest viable tumour area was selected, if also containing the area with the highest tumour grade/highest Ki67; this usually being the same as the block used for Ki67 testing. Tumour size ≥4mm^2 and tumour cellularity percentage ≥10% were also required.

Contributors and included patients per site:

Institution Name (abbreviation)	Included patients	
	n (pN0)	n (pN1(mi))
Oslo University Hospital (OUS)	394	10
Stavanger University Hospital (SUS)	198	5
Vestre Viken Hospital Trust (VV)	193	9
Hospital of Southern Norway (SS)	167	2
University of North Norway (UNN)	154	8
Østfold Hospital Trust (SØK)	155	6
Innlandet Hospital Trust (SI)	154	3

Haukeland University Hospital (HUS)	141	1
St. Olavs Hospital	125	6
Nordland Hospital (NLSH)	105	12
Telemark Hospital Trust (SiT)	111	5
Møre og Romsdal Hospital Trust (SHMR)	95	7
Akershus University Hospital (AHUS)	67	2
Vestfold Hospital Trust (SiV)	74	3
Haugesund Hospital	46	2
Førde Central Hospital	21	1
Levanger Hospital	17	1
