# Supplementary material for: Impact of Prosigna test on adjuvant treatment decision in lymph node-negative early breast cancer—a prospective national multicentre study (EMIT-1)
Source: ESMO Open. 2024 Jun 4;9(6):103475. doi: 10.1016/j.esmoop.2024.103475 (PMC11190479; doi:10.1016/j.esmoop.2024.103475)
Supplement: Supplementary Tables [file mmc2.docx]

Supplementary Tables

**Table S1a.** Systemic adjuvant treatment recommendations for patients with HR+HER2- pN0 EBC in Norway

| BASED ON STANDARD HISTOPATHOLOGY | | | | | |
| --- | --- | --- | --- | --- | --- |
|  |  |  | **CLINICAL LOW RISK** | **CLINICAL INTERMEDIATE RISK** | **CLINICAL HIGHER RISK** |
|  |  |  | NO TREATMENT | ENDOCRINE ALONE | CHEMO + ENDOCRINE |
| LUMINAL A- LIKE | ER≥50% G1-2 and “low” proliferation (Ki67)^#^ | pT1a-b | X |  |  |
|  |  | pT1c G1 | X |  |  |
|  |  | pT1c G2 or pT2 G1 |  | X |  |
|  |  | pT2 G2 |  | X | X* |
| LUMINAL B-LIKE | G2-3 and “high” proliferation (Ki67) ^#^ or ER< 50% | pT1a-b |  | X |  |
|  |  | pT1c-T2 |  |  | X |
| INCLUDING PROSIGNA TEST | | | | | |
|  |  |  | NO TREATMENT | ENDOCRINE ALONE | CHEMO+ ENDOCRINE |
| LUMINAL A | ROR score 0-40 | pT1a-b | X |  |  |
|  |  | pT1cG1 | X |  |  |
|  |  | pT1cG2 |  | X |  |
|  |  | pT2 |  | X |  |
|  | ROR score 41-60 | pT1a-b | X |  |  |
|  |  | pT1c |  | X |  |
|  |  | pT2 |  | X | X* |
| LUMINAL B | ROR score 41-60 | pT1a-b |  | X^1^ | X^2^ |
|  |  | pT1c |  | X | X^2 or^ * |
|  |  | pT2 |  |  | X |
|  | ROR score>60 | pT1a-b |  | X^1^ | X^2^ |
|  |  | pT1c-T2 |  |  | X |

^#^Classification as luminal A-like versus luminal B-like with intermediate Ki67 (10% below to 10% above median) should be considered according to all available analyses. If not otherwise conclusive: >/< median Ki67 for arbitrary classification. * if premenopausal. An alternative to chemotherapy may be optimized endocrine treatment with goserelin. ^1^ER≥50%, ^2^ER<50%. Basal-like and HER2enriched subtypes are treated according to Luminal B subtype. ROR score, Risk of Recurrence score.

The role of chemotherapy (or optimized endocrine treatment) for premenopausal patients were emphasised after the TailorX-study results were published in 2021. Treatment recommendations from study start until 17^th^ June 2021: Table S1b.

**Table S1b.** Systemic adjuvant treatment recommendations for patients with HR+HER2- pN0 EBC in Norway from study start until 17^th^ June 2021

| BASED ON STANDARD HISTOPATHOLOGY | | | | | |
| --- | --- | --- | --- | --- | --- |
|  |  |  | **CLINICAL LOW RISK** | **CLINCICAL INTERMEDIATE RISK** | **CLINCIAL HIGHER RISK** |
|  |  |  | NO TREATMTENT | ENOCRINE ALONE | CHEMO + ENODCRINE |
| LUMINAL A- LIKE | ER≥50% G1-2 and “low” proliferation (Ki67)^#^ | pT1a-b | X |  |  |
|  |  | pT1c G1 | X |  |  |
|  |  | pT1c G2 |  | X |  |
|  |  | pT2 |  | X |  |
| LUMINAL B-LIKE | G2-3 and “high” proliferation (Ki67) ^#^ or ER< 50% | pT1a-b |  | X |  |
|  |  | pT1c-T2 |  |  | X |

| INCLUDING PROSIGNA TEST | | | | | |
| --- | --- | --- | --- | --- | --- |
|  |  |  | NO TREATMENT | ENDOCRINE ALONE | CHEMO+ ENDOCRINE |
| LUMINAL A | ROR score 0-40 | pT1a-b | X |  |  |
|  |  | pT1cG1 |  | X |  |
|  |  | pT1cG2-3 |  | X |  |
|  |  | pT2 |  | X |  |
|  | ROR score 41-60 | pT1a-b | X |  |  |
|  |  | pT1c |  | X |  |
|  |  | pT2 |  | X |  |
| LUMINAL B | ROR score 40-60 | pT1a-b |  | X^1^ |  |
|  |  | pT1c |  | X^1^ | X^2^ |
|  |  | pT2 |  |  | X |
|  | ROR score>60 | pT1a-b |  | X^1^ | X^2^ |
|  |  | pT1c-T2 |  |  | X |

^1^ER≥50%. If ER<50%: consider chemotherapy followed by endocrine treatment. ^2^ER<50%. Basal-like and HER2enriched subtypes are treated according to Luminal B subtype. ROR score, Risk of Recurrence score.

**Table S2.** Characteristics of total study population (including patients with pN0 and pN1(mi) (*n* = 2300)), eligible lymph node negative study population without conclusive Prosigna test (*n* = 35) and patients with conclusive Prosigna test, but no post-treatment decision (*n* = 4)

| CHARACTERISTICS | | TOTAL STUDY POPULATION *n* = 2300 | NOT CONCLUSIVE PROSIGNA  *n = 35* | NO POST-TREATMENT DECISION *n = 4* |
| --- | --- | --- | --- | --- |
|  | | n (%) | n (%) | n (%) |
|  |  |  |  |  |
| Gender | Female | 2287 (99) | 35 (100) | 4 (100) |
|  | Male | 13 (0.6) | 0 | 0 |
| Age (years) | Median (range) | 60 (19-89) | 54 (27-74) | 67 (64-79) |
|  | Categories |  |  |  |
|  | <40 | 80 (3.5) | 3 (8.6) | 0 |
|  | 40-50 | 388 (17) | 7 (20) | 0 |
|  | 51-60 | 780 (34) | 14 (40) | 0 |
|  | 61-70 | 788 (34) | 9 (26) | 3 (75) |
|  | >70 | 264 (12) | 2 (5.7) | 1 (25) |
| T-category | pT1a | 81 (3.5) | 17 (49) | 0 |
|  | pT1b | 490 (21) | 6 (17) | 1 (25) |
|  | pT1c | 1196 (52) | 9 (26) | 1 (25) |
|  | pT2 | 533 (23) | 3 (8.6) | 2 (50) |
| N-category | pN0 | 2152 (94) | 33 (94) | 4 (100) |
|  | pN0(i+) | 65 (2.8) | 2 (5.7) | 0 |
|  | pN1(mi) | 83 (3.6) | - | - |
| Ki67 | Median (range) | 19 (1-92) | 12 (1-60) | 15 (9-29) |
| Histological grade | G1 | 547 (24) | 11 (31) | 1 (25) |
|  | G2 | 1356 (59) | 17 (49) | 3 (75) |
|  | G3 | 395 (17) | 5 (14) | 0 |
|  | Missing | 2 (0) | 2 (5.8) | 0 |
| ER | Positive 1- < 10% | 10 (0.4) | 1 (2.9) | 0 |
|  | Positive 10- < 50% | 38 (1.7) | 0 | 0 |
|  | Positive ≥ 50% | 2237 (97) | 34 (97) | 4 (100) |
|  | Positive, unspecified | 15 (0.7) | 0 | 0 |
| PR | Negative < 10% | 363 (16) | 4 (11) | 0 |
|  | Positive 10- < 50% | 367 (16) | 8 (23) | 0 |
|  | Positive ≥ 50% | 1566 (68) | 23 (66) | 4 (100) |
|  | Positive, unspecified | 4 (0.2) | 0 | 0 |
| PAM50 subtype | Luminal A | 1413 (61) | - | 4 (100) |
|  | Luminal B | 811 (35) | - | 0 |
|  | HER2 enriched | 21 (0.9) | - | 0 |
|  | Basal-like | 20 (0.9) | - | 0 |
|  | Missing | 35 (1.5) | - | 0 |
| ROR-category | 0-40 | 1112 (48) | - | 2 (50) |
|  | 41-60 | 704 (31) | - | 2 (50) |
|  | >60 | 449 (20) | - | 0 |
| ROR score | Median (range) | 41 (0-94) | - | 42 (31-48) |

**Table S3.** Characteristics of HR+/HER2- pN0 patients in Norway 2020-2022 (data from the Cancer Registry of Norway)

| CHARACTERISTICS | CATEGORIES | % OF TOTAL POPULATION |
| --- | --- | --- |
|  |  |  |
| Age (years) | <40 | 2.2 |
|  | 40-50 | 13 |
|  | 51-60 | 27 |
|  | 61-70 | 35 |
|  | >70 | 22 |
| T-category | pT1a | 6.6 |
|  | pT1b | 24 |
|  | pT1c | 46 |
|  | pT2 | 24 |
| Histological grade | G1 | 29 |
|  | G2 | 55 |
|  | G3 | 17 |
|  | Unknown | 0.3 |

**Table S4.** Distribution of intrinsic subtypes and ROR score per age category

|  | INTRINSIC SUBTYPE | | | | ROR SCORE | | |
| --- | --- | --- | --- | --- | --- | --- | --- |
| Age category (years) | Luminal A  *n* (%) | Luminal B  *n* (%) | HER2enriched  *n* (%) | Basal-like  *n* (%) | 0-40  *n* (%) | 41-60  *n* (%) | >60  *n* (%) |
| <40 | 36 (50) | 34 (47) | 0 | 2 (2.8) | 30 (42) | 21 (29) | 21 (29) |
| 40-50 | 230 (63) | 128 (35) | 4 (1.1) | 6 (1.6) | 176 (48) | 121 (33) | 71 (19) |
| 51-60 | 490 (68) | 224 (31) | 4 (0.6) | 6 (0.8) | 400 (55) | 199 (28) | 125 (17) |
| 61-70 | 482 (64) | 263 (35) | 8 (1.1) | 6 (0.8) | 374 (49) | 252 (33) | 133 (18) |
| >70 | 114 (45) | 139 (55) | 2 (0.8) | 0 | 79 (31) | 93 (37) | 83 (33) |

**Table S5.** Treatment decisions pre-Prosigna test, excluding and including patient-related factors (PRF)

| TREATMENT DECISION |  | WITHOUT PRF  *n* (%) | WITH PRF  *n* (%) |
| --- | --- | --- | --- |
| No systemic treatment (NT) | All | 596 (27) | 592 (27) |
|  | ≤50 years | 85 (19) | 83 (19) |
|  | >50 years | 511 (29) | 509 (29) |
| Endocrine treatment alone (ET) | All | 818 (38) | 840 (39) |
|  | ≤50 years | 140 (32) | 145 (33) |
|  | >50 years | 678 (39) | 695 (40) |
| Chemotherapy + endocrine treatment (CT+ ET) | All | 764 (35) | 746 (34) |
|  | ≤50 years | 215 (49) | 212 (48) |
|  | >50 years | 549 (32) | 534 (31) |

% of pN0 study population for all *(n* = 2178) patients and for patients ≤ 50 (*n* = 440) or > 50 (*n* = 1738) years separately.

**Table S6.** Classification of patients according to clinical risk assessment as performed in the MINDACT study*

|  | |  | CLINICAL RISK (MINDACT) | |
| --- | --- | --- | --- | --- |
|  |  | *n* | C-low *n* (%) | C-high *n* (%) |
|  |  | 2178 | 1501 (69)^#^ | 677 (31)^#^ |
| PRE-PROSIGNA TREATMENT DECISION (EMIT*)* | NO CHEMOTHERAPY (%) | 1414 | 1211^!^ (81) | 203 (30) |
|  | CHEMOTHERAPY (%) | 764 | 290(19) | 474^¤^ (70) |
| POST-PROSIGNA TREATMENT DECISION (EMIT) | NO CHEMOTHERAPY (%) | 1657 | 1362 (91) | 296 (44) |
|  | CHEMOTHERAPY (%) | 521 | 139 (9.3) | 382 (56) |

*Modified version of Adjuvant! Online. All tumours are ER positive HER2 negative pN0. No chemotherapy: No systemic treatment (NT) or endocrine
treatment alone (ET). Chemotherapy: Chemotherapy followed by endocrine treatment (CT+ET). ^#^Percent of all patients included. The other percentages represents % of the respective clinical risk group.
**^!^**86% (1211 out of 1414) of the patients assigned to no chemotherapy were clinical low risk.
^#^62% (474 out of 764) of patients assigned to chemotherapy were categorized as clinical high risk.

**Table S7.** Treatment decisions pre- and post-Prosigna test per age category and according to menopausal status

| TREATMENT DECISION |  | PRE-PROSIGNA | | POST-PROSIGNA | |
| --- | --- | --- | --- | --- | --- |
|  | **Age category (years) or menopausal status^#^** | **Without PRF**  ***n* (%)** | **With PRF**  ***n* (%)** | **Without PRF**  ***n* (%)** | **Final treatment decision**  ***n* (%)** |
| No systemic treatment (NT) | <40 | 10 (14) | 9 (13) | 12 (17) | 13 (18) |
|  | 40-50 | 75 (20) | 74 (20) | 68 (19) | 69 (19) |
|  | 51-60 | 227 (31) | 225 (31) | 219 (30) | 221 (31) |
|  | 61-70 | 241 (32) | 241 (32) | 218 (29) | 221 (29) |
|  | >70 | 43 (17) | 43 (17) | 38 (15) | 42 (17) |
|  | Premenopausal | 116 (19) | 114 (19) | 114 (21) | 117 (21) |
|  | Postmenopausal | 451 (76) | 449 (76) | 415 (75) | 423 (75) |
| Endocrine treatment alone (ET) | <40 | 22 (31) | 23 (32) | 27 (38) | 26 (36) |
|  | 40-50 | 118 (32) | 122 (33) | 193 (52) | 187 (51) |
|  | 51-60 | 256 (35) | 260 (36) | 359 (50) | 359 (50) |
|  | 61-70 | 303 (40) | 308 (41) | 400 (53) | 403 (53) |
|  | >70 | 119 (47) | 127 (50) | 123 (48) | 154 (60) |
|  | Premenopausal | 172 (21) | 177 (21) | 268 (24) | 264 (23) |
|  | Postmenopausal | 617 (75) | 634 (75) | 801 (73) | 833 (74) |
| Chemotherapy + endocrine treatment (CT+ET) | <40 | 40 (56) | 40 (56) | 33 (46) | 33 (46) |
|  | 40-50 | 175 (48) | 172 (47) | 107 (29) | 112 (30) |
|  | 51-60 | 241 (33) | 239 (33) | 146 (20) | 144 (20) |
|  | 61-70 | 215 (28) | 210 (28) | 141 (19) | 135 (18) |
|  | >70 | 93 (37) | 85 (33) | 94 (37) | 59 (23) |
|  | Premenopausal | 250 (33) | 247 (33) | 156 (30) | 157 (33) |
|  | Postmenopausal | 497 (65) | 482 (65) | 349 (67) | 309 (64) |

PRF: patient-related factors. At the start, the study eCRF did not distinguish between guideline-alone treatment and final administered treatment. For these cases (*n = 123*), post-Prosigna treatment decision according to guidelines and final treatment decision were considered identical. The results remained unchanged if these patients were excluded (0.1-0.7% absolute difference within the treatment groups). **^#^** Premenopausal includes patients with regular menstruation, menstrual irregularities and hormonal intrauterine device.

**Table S8.** Treatment decision uncertainty

| TREATMENT DECISION |  | PRE-PROSIGNA | |
| --- | --- | --- | --- |
|  | **Age categories (years)** | **Uncertainty without PRF (%)** | **Uncertainty with PRF (%)** |
| No systemic treatment (NT) | All | 12 | 12 |
|  | <40 | 30 | 22 |
|  | 40-50 | 15 | 14 |
|  | 51-60 | 12 | 11 |
|  | 61-70 | 12 | 12 |
|  | >70 | 12 | 12 |
| Endocrine treatment alone (ET) | All | 27 | 28 |
|  | <40 | 46 | 48 |
|  | 40-50 | 33 | 32 |
|  | 51-60 | 28 | 27 |
|  | 61-70 | 27 | 29 |
|  | >70 | 19 | 22 |
| Chemotherapy + endocrine treatment (CT+ET) | All | 36 | 38 |
|  | <40 | 25 | 25 |
|  | 40-50 | 33 | 36 |
|  | 51-60 | 34 | 36 |
|  | 61-70 | 42 | 43 |
|  | >70 | 38 | 44 |

PRF: patient-related factors

**Table S9.** Treatment decisions post-Prosigna test (*n* = 2178)

| TREATMENT DECISION |  | POST-PROSIGNA*  *n* (%) | FINAL DECISION **  *n* (%) |
| --- | --- | --- | --- |
| No systemic treatment (NT) | All | 555 (25) | 566 (26) |
|  | ≤50 years | 80 (18) | 82 (19) |
|  | >50 years | 475 (27) | 484 (28) |
| Endocrine treatment alone (ET) | All | 1102 (51) | 1129 (52) |
|  | ≤50 years | 220 (50) | 213 (48) |
|  | >50 years | 882 (51) | 916 (53) |
| Chemotherapy + endocrine treatment (CT+ET) | All | 521 (24) | 483 (22) |
|  | ≤50 years | 140 (32) | 145 (33) |
|  | >50 years | 381 (22) | 338 (19) |

*Guideline-based treatment decision. **Actual treatment started. % of pN0 study population for all (*n* = 2178) patients and for patients ≤ 50 (*n* = 440) or > 50 (*n* = 1738) years separately. Excluding patients with ER expression <10% (*n* = 9) did not change the distribution of treatment decisions.

**Table S10.** Treatment alterations, including patient-related factors

| TREATMENT DECISION ALTERATION | ALL PATIENTS (*n=2178*) | | | PATIENTS ≤ 50 YEARS (*n=440*) | | | PATIENTS > 50 YEARS (*n=1738*) | | |
| --- | --- | --- | --- | --- | --- | --- | --- | --- | --- |
|  | PRE-PROSIGNA DECISION | | | PRE-PROSIGNA DECISION | | | PRE-PROSIGNA DECISION | | |
|  | NT, *n* (%) | ET, *n* (%) | CT+ ET, *n* (%) | NT, *n* (%) | ET, *n* (%) | CT+ ET, *n* (%) | NT, *n* (%) | ET, *n* (%) | CT+ ET, *n* (%) |
| CT→ET | - | - | 342 (46) | - | - | 87 (41) | - | - | 255 (48) |
| CT→NT | - | - | 7 (0.9) | - | - | 1 (0.5) | - | - | 6 (1.1) |
| ET→NT | - | 72 (8.6) | - | - | 14 (9.7) | - | - | 58 (8.3) | - |
| UNCHANGED | 487 (82) | 687 (82) | 397 (53) | 67 (81) | 111 (77) | 124 (59) | 420 (83) | 576 (83) | 273 (51) |
| NT→ET | 100 (17) | - | - | 15 (18) | - | - | 85 (17) | - | - |
| NT→CT | 5 (0.8) | - | - | 1 (1.2) | - | - | 4 (0.8) | - | - |
| ET→CT | - | 81 (9.6) | - | - | 20 (14) | - | - | 61 (8.8) | - |
| TOTAL | **592** | **840** | **746** | **83** | **145** | **212** | **509** | **695** | **534** |

CT: Chemotherapy. ET: endocrine therapy. NT: no systemic treatment

**Table S11.** Clinical profiles and molecular subtypes for patients with pT1c-T2 pN0, sites with more than 50 pts included

|  |  | NO CHEMO CANDIDATES | UNCERTAIN CHEMO CANDIDATES | CHEMO CANDIDATES |
| --- | --- | --- | --- | --- |
|  |  | *n* (total %; Range) | *n* (total %; Range) | *n* (total %; Range) |
| Total |  | 318 (100) | 612 (100) | 574 (100) |
| PAM50 subtype | Luminal A | 299 (94; 71-100) | 422 (69; 57-91) | 121 (21; 7.4-46) |
|  | Luminal B | 19 (6.0; 0-29) | 190 (31; 9.1-44) | 421 (73; 52-93) |
|  | HER2 enriched | 0 | 0 | 15 (2.6; 0-6.9) |
|  | Basal-like | 0 | 0 | 17 (3.0; 0-14) |
| ROR score | Median (range) | 26 (0-70) | 41 (8-87) | 61 (9-94) |
|  | 0-40 | 275 (87; 52-100) | 291 (48; 34-69) | 57 (10; 3.7-30) |
|  | 41-60 | 41 (13; 0-48) | 257 (42; 23-53) | 215 (38; 19-57) |
|  | >60 | 2 (0.6; 0-6.3) | 64 (11; 0-16) | 302 (53; 24-78) |

No chemo candidates: G2 and Ki67< 0.5x local laboratory median score or G1 and Ki67<1x local laboratory median score. Uncertain chemo candidates: G2 and Ki67 0.5-1.5 x local laboratory median score. Chemo candidates: G2 and Ki67>1.5 x local laboratory median score or G3 and Ki67>1x local laboratory median score. Range: Range in proportion across sites.
