# Supplementary figures and images for: Impact of Prosigna test on adjuvant treatment decision in lymph node-negative early breast cancer—a prospective national multicentre study (EMIT-1)
Source: ESMO Open. 2024 Jun 4;9(6):103475. doi: 10.1016/j.esmoop.2024.103475 (PMC11190479; doi:10.1016/j.esmoop.2024.103475)

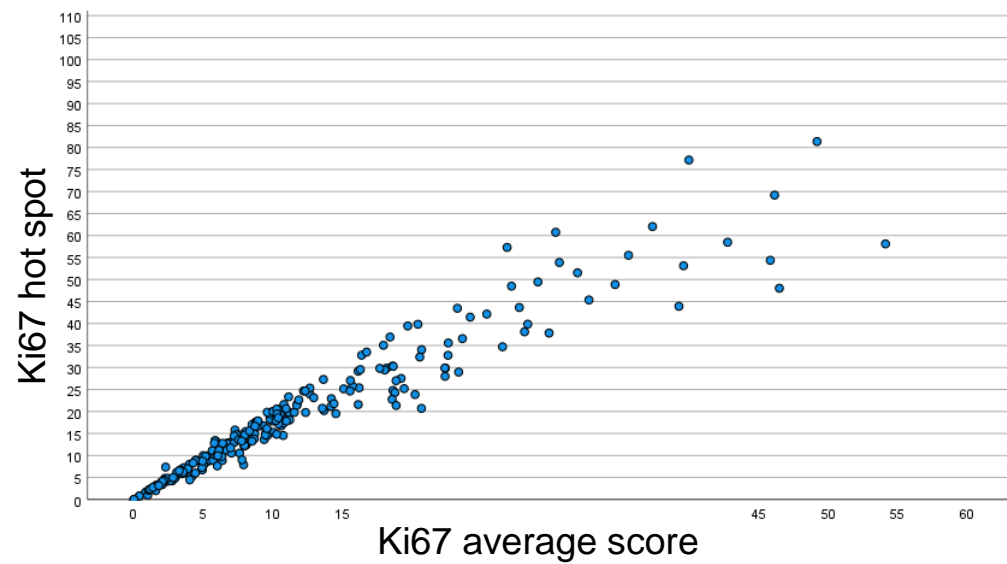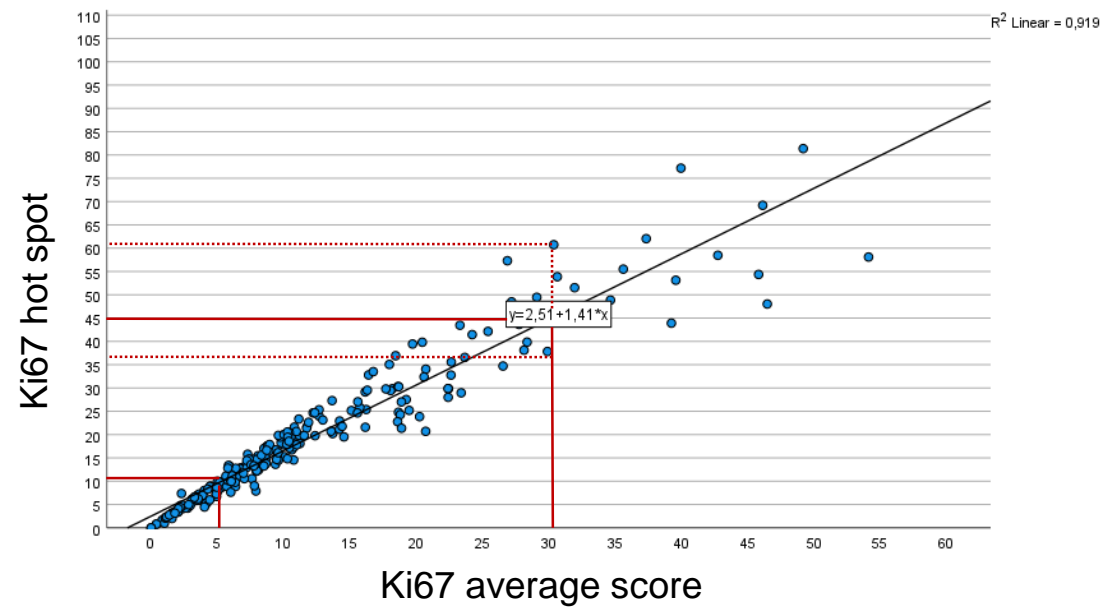

Based on unpublished data from the Oslo-1 study, Ohnstad et al 2017

Supplement: Supplementary Figure S1 [file mmc3.pdf]

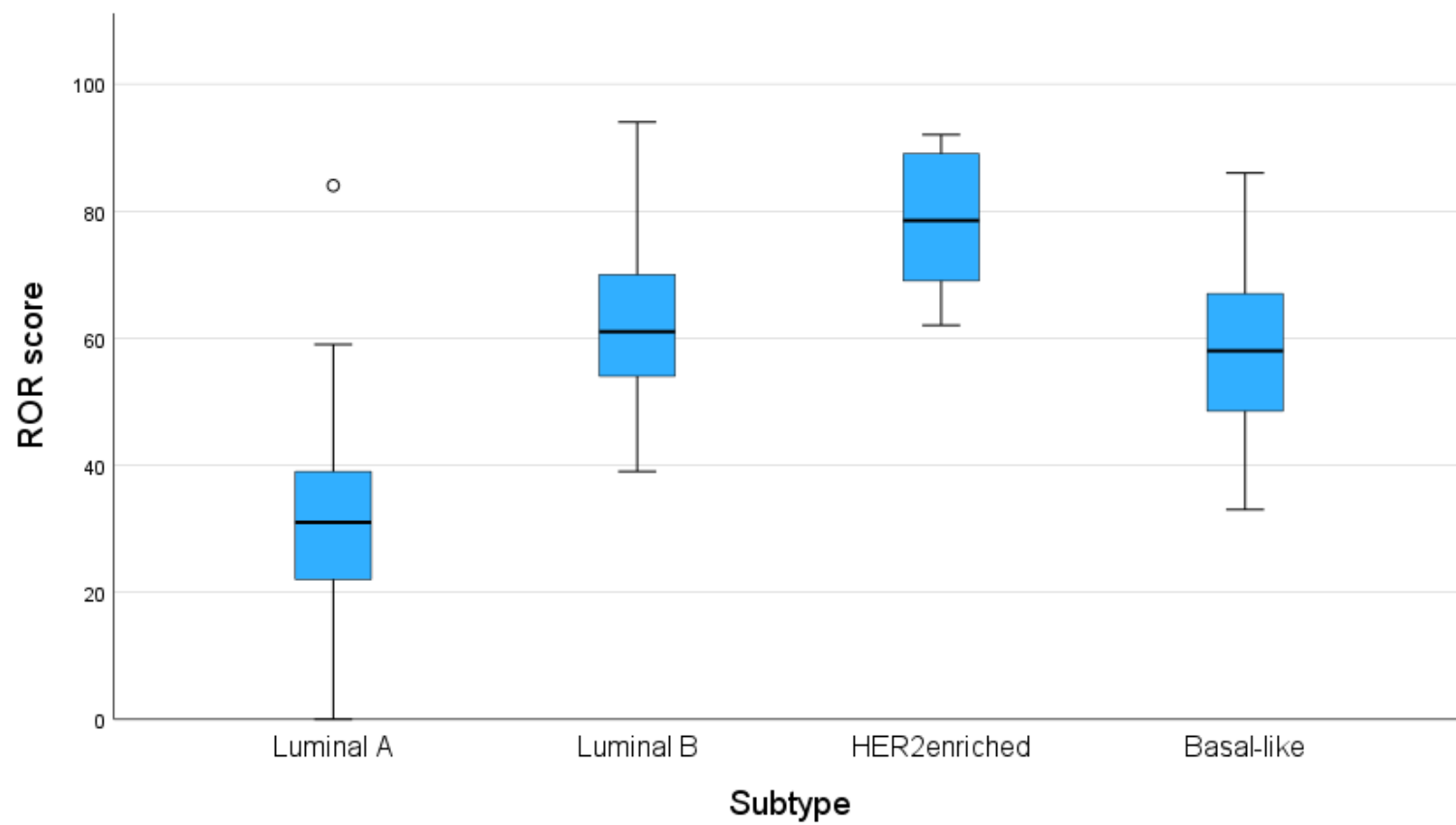

Supplement: Supplementary Figure S2 [file mmc4.pdf]

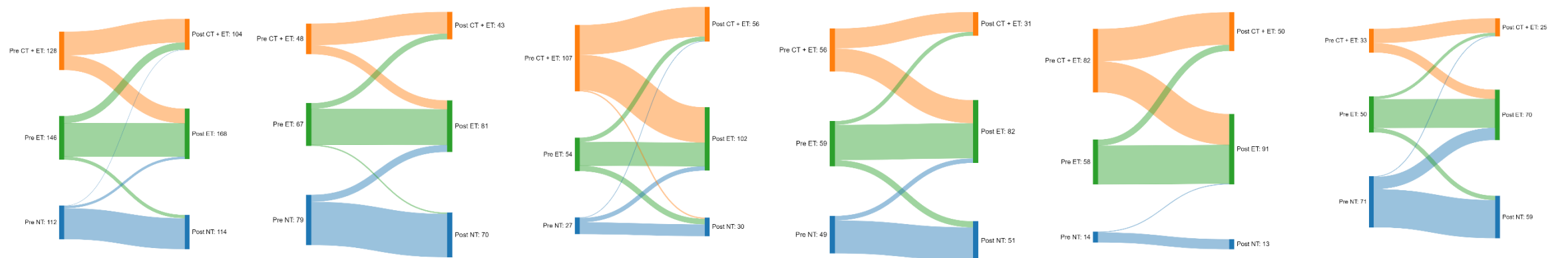

Site

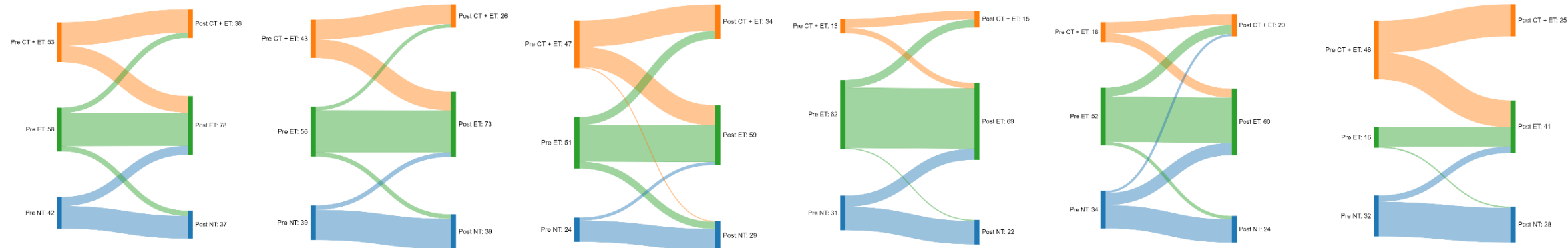

Site

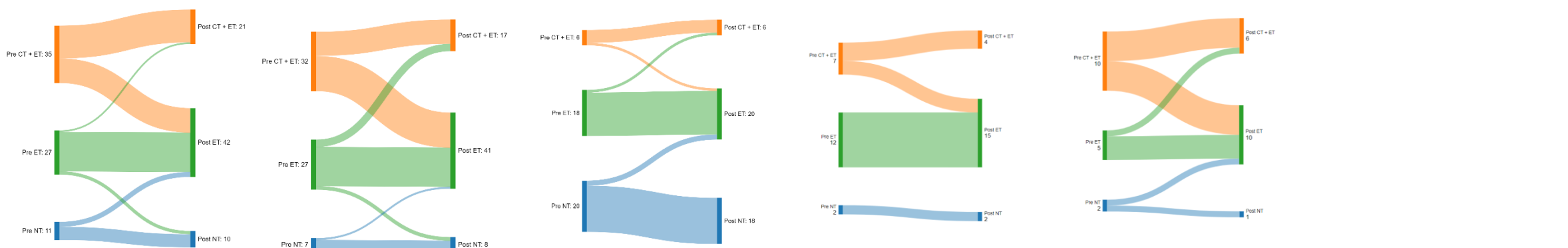

Site

Supplement: Supplementary Figure S3 [file mmc5.pdf]

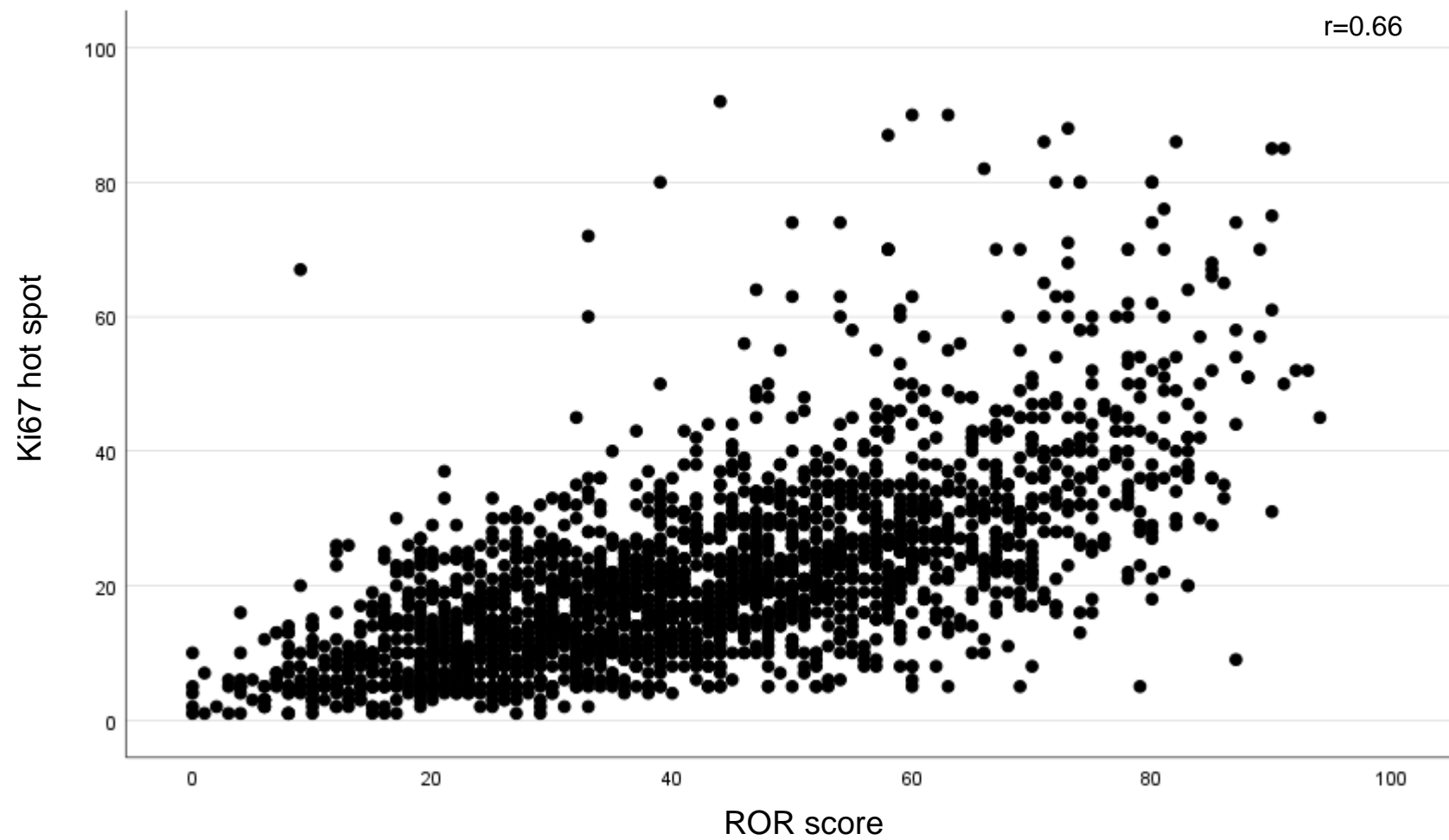

Supplement: Supplementary Figure S4 [file mmc6.pdf]

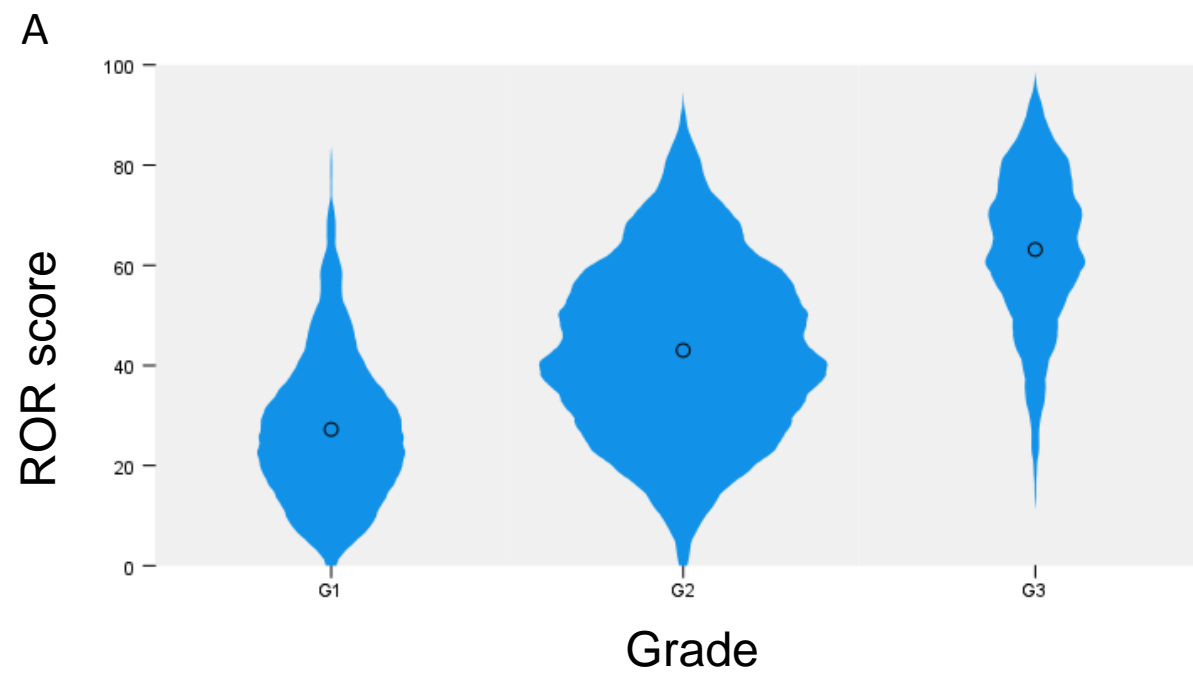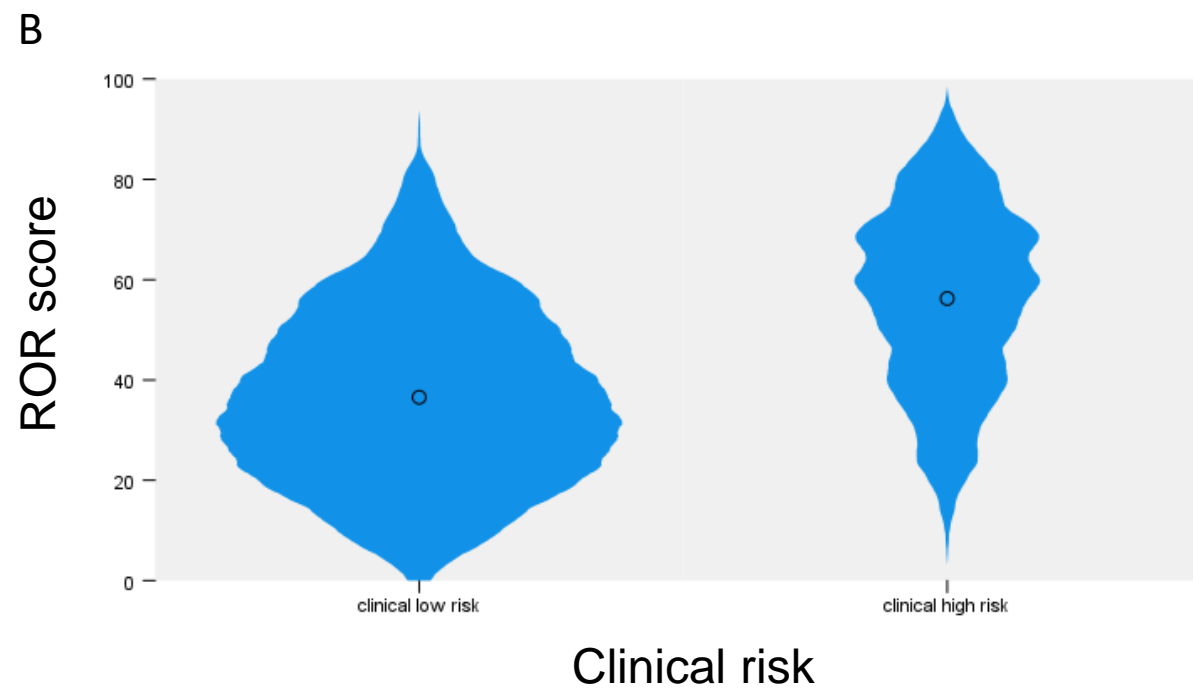

Supplement: Supplementary Figure S5 [file mmc7.pdf]
