# Supplementary material for: Impact of Prosigna test on adjuvant treatment decision in lymph node-negative early breast cancer—a prospective national multicentre study (EMIT-1)
Source: ESMO Open. 2024 Jun 4;9(6):103475. doi: 10.1016/j.esmoop.2024.103475 (PMC11190479; doi:10.1016/j.esmoop.2024.103475)
Supplement: Supplementary data [file mmc8.pdf]

Establishment of **M**olecular profiling for **I**ndividual clinical routine **T**reatment decision in Early Breast Cancer (EMIT^{EBC}-1)

Protocol version: 3.2, 09.May 2023

Sponsor: Oslo University Hospital;

Postbox 4953, Nydalen, 0424 Oslo, Tel. +47 22 93 40 00

Multicenter trial planned to include patients from all Norwegian breast cancer units interested to participate.

Participating hospitals: See appendix 1.

Principle Investigator: Professor Bjørn Naume, MD PhD, OUS.

On behalf of the Steering committee, it is declared that version 3.2 (09.May 2023) is the current version of this study protocol.

Bjørn Naume, PRINCIPAL INVESTIGATOR 09.05.2023

Table of Contents

1. STUDY SYNOPSIS....4

2. BACKGROUND AND RATIONALE....7
   2.1 Introduction....7
   2.2 The current analysis and treatment of breast cancer....8
   2.2.1 Routine immunohistochemical/histopathological analysis....8
   2.2.2 Endocrine treatment....9
   2.2.3 Chemotherapy....9
   2.3 Redefining breast cancer....10
   2.3.1 Multi-parameter tests vs single marker analyses....11
   2.4 Breast cancer treatment-related side effects....12
   2.5 Health resource use....13
   2.6 Relevant results....13
   2.7 Resources and collaborative basis for the study....16
   2.8 Rationale....17

3. OBJECTIVES OF THE STUDY....18

4. STUDY DESIGN....18
   4.1 Surgery....21
   4.2 Scheduled visits/follow-up....21

5. STUDY POPULATION....22
   5.1 Target population....22
   5.2 Inclusion criteria....22
   5.3 Exclusion criteria....22

6. SCHEDULE OF ASSESSMENTS, PROCEDURES AND FOLLOW-UP....23
   6.1 Screening....23
   6.2 Procedure and follow-up plan after inclusion to the study....24
   6.3 Study assessments....24
   6.4 Patient related procedures....25
   6.4.1 Tumor tissue sampling....25
   6.4.2 Blood sampling....25
   6.4.3 Patient reported assessments....25
   6.4.4 Health economic assessments....25
   6.5 Primary tumor analyses....25

6.5.1 Analysis of HER2, ER/PgR, Ki67 and Grade 26
6.5.2 Molecular-based multi-parameter analysis, Prosigna™ 26
6.5.3 Other molecular analyses of tumor tissue 27

7. STATISTICAL CONSIDERATIONS AND ANALYTICAL PLAN 27
7.1 Determination of patient number / sample size 27
7.2 Statistical analysis of study data 28

8. ETHICS AND GENERAL STUDY ADMINISTRATION 29
8.1 Ethics 29
8.2 Study organization 29
8.3 Monitoring 30
8.4 Case Report Form and Data management 31
8.5 Archiving 31
8.6 Inspections 31
8.7 Confidentiality of trial documents and subject records 32
8.8 Publication 32

9. REFERENCES 32

10. Appendix 1: Participating hospitals 41

11. Appendix 2: Protocol history 42

1. STUDY SYNOPSIS

“Establishment of Molecular profiling for Individual clinical routine Treatment decision in Early Breast Cancer (EMIT^{EBC}) “

The present project focuses on how to reduce both over- and undertreatment with adjuvant chemotherapy to a large number of breast cancer patients in Norway. A set of primary tumor prognostic factors can be analyzed for potential achievement of this. Furthermore, multi-parameter tests, including detailed molecular analysis of the primary tumors, might further improve the selection of patients among the lymph node negative. The study seeks to advance the development of personalized treatment of patients with mainly lymph node negative early breast cancer, by the evaluation of multi-parameter analysis as a means of identifying those patients who are likely to benefit from chemotherapy whilst sparing those who are unlikely to do so from an unnecessary and unpleasant treatment.

Objectives

- To establish and optimize the use of molecular-based multi-parameter assay (i.e. Prosigna test) for adjuvant treatment decision in patients with hormone sensitive primary breast cancer without lymph node (macro)metastasis, compared to decisions based on standard practice. Differences in the number of patients receiving the various treatments using test-directed (Prosigna) treatment compared to standard adjuvant treatment recommendations will be registered.

- To determine recurrence-free interval and distant recurrence-free interval after 5 years of follow-up (8 years from study start) for the lymph node negative patients.

- To identify differences in reported quality of life (QoL), late effects and working ability/capacity among patients receiving chemotherapy compared to those without chemotherapy.

- To establish the cost-effectiveness (i.e. health resource use in hospitals and society) of test-directed (Prosigna) treatment strategy compared to standard practice.

- To investigate the feasibility of the molecular classification-based treatment and achieve integrated knowledge on the consequences of nation-wide implementation of a molecular multi-parameter test.

Study design

The study will be run as a one-armed multi-center trial. All patients with hormone receptor positive, HER2 negative, pT1-2 tumors and clinically node negative status will be informed at first postoperative

visit. In addition, patients with tumor size ≤20mm and lymph node micrometastases only may also be informed. Included patients will be asked to complete quality of life questionnaires (QoL) including health resource use, late effects and work ability.

After inclusion, the patients will be followed for breast cancer related events and QoL measurements until 5 years. Further follow-up will be performed according to standard recommendations.

Target Population

The study will include pN0/pN1mi patients with primary non-metastatic, hormone receptor positive, HER2 negative breast cancer as defined below.

Inclusion Criteria

1. Written informed consent (informed consent document approved by the Independent Ethics Committee [IEC]) obtained prior to any study-specific procedure.
2. Female or male age ≥ 18 years.
3. Able to comply with the protocol.
4. Primary surgery completed or if re-resection needed, a change from pT1 to pT2 categorization is not expected.
5. Histologically confirmed adenocarcinoma of the breast ≤ 5.0 cm in size (pT1-2) without metastasis to regional lymph nodes (pN0) or ≤ 20 mm in size (pT1) with lymph node micrometastases only (pN1mi).
6. Primary tumor concluded as hormone receptor positive (ER ≥ 1%), HER2 negative.

Exclusion Criteria

1. Metastasis to regional lymph nodes or distant sites/organs. Lymph node micrometastases (pN1mi) is allowed if pT1 (confer subsection 5 above).
2. Previous treatment for localized breast cancer. Previous treatment for DCIS is allowed.
3. HER2 positive.
4. ER negative tumor (< 1% expression).
5. Other concomitant or earlier carcinoma less than five years prior to the breast cancer diagnosis, except for basal cell carcinoma and *in situ* cervix cancer.
6. Use of or participation in intervention trials testing treatment with any investigational anti-cancer drug. Participation in other types of intervention trials is allowed (such participation needs to be registered).
7. Evidence of any other disease or condition that by the investigator is considered to impede follow-up of the patients.

Statistical Consideration

In the retrospective EMIT0 study, including an equivalent study population, the breast cancer specific death at 15 years follow-up was 4% for patients with low risk ROR score (Ohnstad et al, BCR 2017). Following the anticipated reduced and altered use of chemotherapy by including Prosigna in adjuvant treatment decision, the current project aims to retain excellent survival for the patient group not undergoing chemotherapy, a group expected to increase in size compared to decisions based on conventional histopathological assessments. With inclusion of close to 2150 lymph node negative patients (the patient group to be included in the survival analyses) during a period of approximately 3-3.5 years, it is estimated that 1500 patients will not be recommended chemotherapy. If the distant recurrence-free interval is 96%, statistical analysis reveal a 95% confidence interval for distant recurrence free interval of 95-97% with this sample size of chemotherapy un-treated patients. The primary analyses are planned 8 years after study start. Patients who participate in an allowed interventional trial (see exclusion criteria) in parallel, should be marked in order to perform separate analyses without these patients included.

2. BACKGROUND AND RATIONALE

2.1 Introduction

The majority of patients with breast cancer are diagnosed in the early stage of the disease and with no evidence of axillary node metastasis. Overall, the prognosis for node negative breast cancer is good [1-3]. Several known primary tumor factors are used to identify patients with breast cancer who are recommended adjuvant systemic treatment after completion of primary surgery to reduce the risks of relapse and death [4, 5]. However, a large majority of patients with early stage breast cancer with relatively small primary tumors and no axillary lymph node metastasis receive chemotherapy without being at significant risk of developing recurrent distant disease. Despite a large number of studies that have addressed the prognostic impact of primary tumor factors by routine histopathology, also combined with factors to improve the prognostic information [6-10], current routine diagnostic analyses are not able to provide the clinician with sufficiently reliable information to make clear treatment recommendations on chemotherapy use for large number of patients. To improve decision-making in breast cancer, there is a need for a more accurate classification of patients into prognostic groups.

Overtreatment includes major concerns about side effects and risk/benefit ratio for a large number of patients. In the most favorable prognostic group that receives adjuvant systemic therapy today, less than 5% of the patients would potentially benefit from the treatment. Side effects, however, affect the majority of the patients, although to a variable degree [11, 12]. In addition to undesirable toxicities and economic disadvantages for the individual patient, the cost for the society includes an increased number of disabled individuals resulting in increased social security expenses, and unsatisfactory use of health care resources, both related to drugs and personnel. For the future, it is critical to reduce the overtreatment as much as possible, in order to use the treatment resources only where a reasonable improvement in clinical outcome can be obtained.

Whilst estrogen receptors and Human Epidermal Growth Factor Receptor 2 (HER2) expression are used to determine sensitivity to endocrine therapy and trastuzumab respectively, no similar tests exist for chemotherapy sensitivity. Thus, a major focus of research in recent years has been to develop tests of sensitivity to chemotherapy so that patients who would not benefit from such treatment could avoid unpleasant side effects and the society could be spared unnecessary costs. A number of 'multi-parameter' prognostic tests for breast cancer have been developed using molecular techniques. The majority of the assays has been developed primarily as prognostic tests and provides superior prognostic information to conventional assessed histopathological analyses [13-17].

This study, EMIT-1, aims to assess the value of one of these multi-parameter tests, Prosigna, in women and men aged 18 or older who have node negative, pT1-2 tumors that are hormone receptor positive, HER2 negative, and to gain experience from the use of the test in patients with pT1 tumors having only micrometastatic spread to axillary lymph nodes. The assumption behind the study is that new

molecular-based multi-parameter tests improve the stratification of breast cancer patients, allowing for identification of a sizeable subgroup of women with breast cancer in whom chemotherapy offers toxicity without a clinically meaningful benefit. On the other hand, there might also be a subgroup of patients with clinically low-risk, genomically high-risk tumors for whom chemotherapy may be beneficial.

2.2 The current analysis and treatment of breast cancer

The treatment of primary breast cancer, which is undertaken with curative intent, is divided into local (surgery and radiotherapy) and systemic (chemotherapy, endocrine treatment and HER2- targeted drugs) therapies. The goal of systemic treatment is to eliminate occult microscopic metastatic disease and thus prevent incurable distant relapse. Decisions on adjuvant treatment depend on an individual patient's risk of developing future overt metastatic disease. The risk is affected by tumor stage (size and number of involved axillary lymph nodes) and by tumor biology. Relevant biological features include tumor grade, Ki67 and its estrogen receptor (ER) status and HER2 status (see section 2.2.1). These latter two also predict sensitivity to anti-estrogen treatment and HER2-targeted therapy respectively. Distant relapse, which affects a minority of patients, typically occurs after an interval of several years; later relapse is a feature of both ER positive and lower grade tumors [18].

Although male breast cancer is comparatively rare and therefore much less studied, current knowledge does not find the disease fundamentally different from female breast cancer and the treatment is principally the same.

2.2.1 Routine immunohistochemical/histopathological analysis

The traditional classification of breast cancer is based on morphology and immunohistochemical analysis in combination with histopathology and has resulted in improved classification of breast carcinomas. Several markers and examinations have been included in Consensus recommendations, for use in decisions of adjuvant systemic treatment [5, 19, 20]. For hormone receptor positive, HER2 negative (HR+HER2-) patients, representing about 80% of the breast cancer population [21], chemotherapy decision in Norway is based mainly on primary tumor assessment of histologic grade (with three morphological features) and Ki67 protein expression. Although presence of histological Grade 3 is established as a poor prognostic factor, the large histological Grade 2 group biologically seems to constitute a mixture of Grade 1 and Grade 3 tumors [22]. Further subclassification of especially Grade 2 breast tumors, is therefore of importance [22, 23]. The Ki67 results are presented in the pathology report as percentage of positively stained tumor cells and the analysis has been included as a useful marker to estimate proliferation status [5, 20]. Two recent meta-analyses have reported a statistically significant association between high Ki67 expression and increased risk of breast cancer relapse and death [24-26]. Many of these studies however, use different cut-off points between high and low Ki67

expression [27-30]. In order to improve standardization, recommendations for Ki67 assessment have recently been published [31]. Initially, the Ki67 cut-off set by the St. Gallen recommendation was < vs. ≥14%, based on the Ki67 level that can differentiate between luminal A and luminal B breast cancer subtypes [32, 33].

However, in line with international reports, results from the Norwegian Breast Cancer registry (NBCR) have shown that these parameters are hampered with substantial inter-laboratory variations [21], and both suffer from a large "grey-zone" category (Grade 2 and Ki67 15-30% expression) where decisions on chemotherapy use are insecure and clearly unsatisfactory [34, 35]. Furthermore, delineating universal Ki67 cut-off values for chemotherapy recommendations has not been successful [14, 31] and international treatment guidelines differ in acceptance of Ki67 as a biomarker for adjuvant treatment decisions [35, 36]. It has become clear that both histological grade and Ki67 are not sufficiently reliable for use in national treatment guidelines for chemotherapy use. The consequence of current practice is therefore unwanted national variability as well as variability between physicians in adjuvant chemotherapy treatment decisions. There is a strong need for a more precise and robust categorization into risk groups in order to both identify the large group of patients with an excellent outcome with questionable benefit of adjuvant treatment, as well as the smaller group of patients with very high risk of future metastasis.

2.2.2 Endocrine treatment

Endocrine therapy with tamoxifen (if premenopausal) and more recently aromatase inhibitors (AIs; if postmenopausal) is considered to be the mainstay of treatment for women with ER positive disease, the commonest presentation of breast cancer. In addition, endocrine therapy makes a greater contribution to improvements in outcome than does chemotherapy in the overall population [37]. AIs have been shown to be superior to tamoxifen in a number of large randomized clinical trials and current Norwegian Breast Cancer Group (NBCG) guidelines recommend that these drugs should be offered to the majority of postmenopausal patients as well as in combination with ovarian suppression for a subgroup of premenopausal patients [38-40].

2.2.3 Chemotherapy

In recent years, there has been a large expansion in the use of adjuvant chemotherapy in the treatment of early breast cancer to reduce the risks of relapse and death. The Oxford Overview meta-analysis of adjuvant chemotherapy trials suggests that the reduction in the relative risk of relapse and death is similar for all breast cancers, but the absolute benefit is greater for those at highest risk (either from having involved axillary lymph nodes and/or large tumor size)[41]. In Norway as in many other countries, it has become standard to offer chemotherapy with anthracyclines and/or taxanes to many women, also without high risk factors such as axillary node involvement.

Several computerized tools have been developed to aid adjuvant therapy decision-making, particularly for chemotherapy. All of these tools use individual patient and pathological data combined with population data to assess baseline risk. Clinical trial efficacy data is then used to predict individual patient treatment benefit. The best known of these tools are PREDICT [42] and Adjuvant! [43], both recommended in several guidelines, including the Norwegian guideline [40].

However, both PREDICT and Adjuvant! refine existing practice rather than offering a fundamentally new approach to selecting patients who are likely to benefit from chemotherapy.

2.3 Redefining breast cancer

Tumor aggressiveness and therapeutic response may not only be associated with the disturbance of single genes, but rather with the combined influence of many genes. High throughput molecular profiling approaches may be more informative, sensitive and specific than single markers to reflect the actual heterogeneity of the breast cancer disease. It is therefore hypothesized that such technologies will be found more reliable for screening purposes, for diagnostics and prognostication of individual cases, for prediction of therapeutic responses and also convenient for discovering new therapeutic targets.

Since 2000 with the invention of the technology of microarray profiling, a new molecular classification of breast cancer has been developed [44, 45]. This classification divides breast cancers into four main "intrinsic subtypes": Luminal A, luminal B, HER2-enriched and basal-like. These subtypes differ markedly in their clinical behavior and response to therapy. This goes some way to explaining the highly heterogeneous clinical behavior of the disease. Within the intrinsic subtypes, luminal A breast cancer has a significantly better prognosis than the other subtypes. Most breast cancers with a lower proliferation rate (typically Grade 1 or a subgroup of Grade 2) that are both strongly positive for ER expression and which express HER2 at normal levels will fall into the luminal A category [44, 45]. In order to be applicable for routine analysis, further development of this analytical principle resulted in an assay using a reduced gene set built into a classifier termed PAM50 [46-48]. Based on proliferation genes included in the PAM50, a prognostic model, risk of recurrence score (ROR) was developed to estimate clinical outcome. The PAM50 ROR analysis is now available as a standardized FDA approved, CE marked assay termed Prosigna™ using digital bar code technology (NanoString Technologies Inc., subsequently transferred to Veracyte Inc.) that can be performed on formalin-fixed paraffin embedded tissue [49]. In addition, several other molecular-based multi-parameter analytical tests and algorithms have been established the last years, and the prognostic impact has been established, many with strong evidence from large clinical trials (i.e. MammaPrint, Breast Cancer Index (BCI), Oncotype DX recurrence score (RS), Prosigna, EndoPredict (EpClin))[13, 50-52]. The MammaPrint and the Rotterdam signature have identified a 70 gene and 76 gene signatures, respectively, based on microarray technology, which may be used as a tool for decision-making in the treatment of certain subtypes of breast cancer [53-55]. The OncotypeDx technology use expression of 21 genes (including 5 reference genes) measured by RT-PCR to

calculate a recurrence score. Depending on the score (low, intermediate, high) this analysis has been reported to differentiate between patients with no benefit (low/intermediate score) or clear benefit (high score) from use of adjuvant chemotherapy in ER positive breast cancer patients [56]. The recurrence score has been validated and FDA approved for use in selection of chemotherapy to ER positive early breast cancer patients, thus also giving predictive information. However, the data supporting the predictive value was initially based on small patient cohorts [57] and OncotypeDx taken alone was only able to predict risk of recurrence within 5 years of prognosis [58]. On the other hand, all of the tests provide superior information to conventional tumor grade. Some of the tests have been or are being evaluated prospectively in ongoing clinical trials of treatment decisions (TailorX, MINDACT, RxPonder, OPTIMA), and recently both TailorX and MINDACT have reported evidence supporting the omission of chemotherapy for "genomic low/intermediate risk" patients [13, 59-61]. Oncotype DX, EndoPredict and Prosigna have been included in the ASCO, ESMO and NCCN guidelines, as tools to identify patients expected to have no/little benefit from chemotherapy.

To further extend the exploration of expression levels to transcriptional regulation, microarrays on microRNA, chromatin immunoprecipitation (CHIP) of promoter regions, as well as whole genome DNA methylation analysis [62] will give additional insight. Furthermore, SNP analyses/whole genome sequencing of the tumors may be able to refine the classification of the subclasses of breast cancer. Altogether, these high-throughput technologies will give us information on the expression/DNA signature in a particular biopsy/tumor specimen, and also suggestions on how the expression of certain tumor markers, oncogenes, or tumor suppressor genes is regulated and how this affects prognosis.

2.3.1 Multi-parameter tests vs single marker analyses

Previous adjuvant chemotherapy trials in breast cancer have made the assumption that breast cancer is a single entity and that the proportional benefits of chemotherapy apply uniformly to all cancers irrespective of histological characteristics of the tumor. Molecular profiling analyses have resulted in growing knowledge of biologically different breast cancer subclasses.

Consequently, it is necessary to investigate the appropriate use of chemotherapy within the different subtypes.

Evidence that chemotherapy response is influenced by tumor biology comes from analysis of response to pre-surgical (neo-adjuvant) chemotherapy. Analysis of the outcome of treatment according to intrinsic subtype of individual tumors is particularly striking with a pathological complete response rate of 6% in luminal tumors compared to 45% in basal-like type [63]. Two independent studies showed that the chances of achieving a pathological complete response for patients with luminal B tumors were more than the double of the chances for patients with luminal A tumors [48, 64].

Although a combination of immunohistochemical markers has improved classification of breast carcinomas, lack of validity and reproducibility is still an issue of concern [14, 31]. Sotiriou et al concluded that the common genes/pathways upregulated in the molecular multi-parameter profiles,

were related to proliferation [53]. However, the use of Ki67 as a single proliferation marker has not shown superior evidence as surrogate marker.

Nevertheless, all of the above mentioned multi-parameter tests provide superior information to conventional tumor grade. Furthermore, several studies have shown that the standardized PAM50 ROR assay is superior to conventional parameters, immunohistochemical (IHC)-based assays and other multi-gene expression tools [17, 52, 65, 66]. Data from the TransATAC study, comparing Clinical Treatment Score, IHC4, RS, EPclin, BCI and Prosigna, indicate that EPclin and Prosigna score are the strongest predictors of distant recurrence, both in node positive- and node negative, HR+ breast cancer patients. In particular, these markers are promising as identifiers of patient at low-risk of distant recurrence [15]. Delahaye et al recently reported an ultra-low/indolent signature based on the 70-gene signature, identifying a small subgroup of adjuvantly untreated patients with 100% 15 years BCSS [67]. Comparison of this ultra-low signature with the low risk ROR score would be of interest. However, despite potential for improved standardization, many of these multi-parameter analyses so far need fresh tumor material and they are all expensive, thus restricting the clinical use of these tests in most of the global oncology community. The Prosigna test and EndoPredict, on the other hand, are the only tests to date that is recommended used on standard routine formalin fixed paraffin embedded tumor material and can be established in local/regional laboratories at reduced costs.

2.4 Breast cancer treatment-related side effects

Although undoubtedly highly effective for some, there are major concerns about the acute and late side effects of chemotherapy and its risk/benefit ratio for a large number of patients. In the best prognostic group that currently receives adjuvant chemotherapy, 2-5% of the patients would potentially benefit from this treatment (based on a relative risk reduction of 1/3). In contrast, a majority of the patients experience treatment-related side effects, although to a variable degree. This includes hair loss, risk of febrile neutropenia, nausea, painful mouth ulcers, diarrhea or constipation, weight gain, muscle pain, loss of sensation in hands and feet, fatigue and sexual dysfunctions. There is also a small long-term risk of treatment induced leukemia and cardiomyopathy. Most patients are unable to work during and for some time after treatment, which has a considerable cost to society. Many are left with anxiety, fatigue and depression, which severely affect their quality of life for months or even years afterwards. Chronic fatigue (CF) is one of the most common and disabling adverse effects in breast cancer survivors, affecting more than 25% of patients for years after end of treatment [68]. Patients have higher risk of developing severe fatigue if treated with chemotherapy compared to no such treatment [69, 70]. Other late effects in breast cancer survivors include cognitive and psychological complaints, musculoskeletal symptoms and pain, and cardiovascular dysfunction/disease which negatively can affect working capabilities and fatigue. For the most commonly used chemotherapy regimens, increased mortality (0.6%), systolic dysfunction (5%), and congestive heart disease (10% in elderly, as compared to treatment without anthracyclines) has been reported [32]. For the future, it is critical to reduce the overtreatment as much

as possible, in order to use the treatment resources only where a reasonable improvement in clinical outcome can be obtained. In addition, low risk groups (by standard classification) recommended no adjuvant systemic treatment or endocrine treatment may also include patients with high-risk features, causing unwanted undertreatment of some patients.

2.5 Health resource use

In addition to the undesirable toxicities, chemotherapy overtreatment may cause economic disadvantages for individual patients as well as cost to society, including an increased number of disabled individuals with associated social security expenses, and increased use of health care resources, both related to drugs and personnel costs. There is a lack of knowledge about the detailed health economic effects of the use of molecular profiling from prospective clinical trials. Our preliminary estimate suggests that the cost of Prosigna testing for patients with node negative disease will be lower than the expenses for hospitals and society directly related to chemotherapy treatment and associated sick leave expenditure. A 15% reduction in the use of chemotherapy for all HR+HER2- lymph node negative patients should result in an annual saving of 8-13 mill NOK, excluding personal expenses, expenses related to hospitalization due to treatment toxicities and any long-term disability costs. This however requires further investigation with prospective data collection, which will be a focus for this study.

2.6 Relevant results

As mentioned (section 2.3), the PAM50 ROR analysis is now available as a standardized FDA approved, CE marked assay termed Prosigna™ using digital bar code technology (NanoString Technologies Inc., subsequently transferred to Veracyte Inc.). The test has been used in several large retrospective clinical trials (section 2.3) including the Danish study analyzing tumor tissue from 2749 patients, showing improved prediction of clinical outcome, over and above what can be achieved from standard clinical and pathological variables as well as reliable identification of patients that may be spared chemotherapy [71].

In Norway, a retrospective study of 653 early breast cancer patients with available standard formalin-fixed paraffin embedded (FFPE) primary tumor tissues and long-term follow-up (median 16 years) (Oslo 1 study), was used for validation of the Prosigna test[72]. Multivariate analysis showed that the PAM50 intrinsic subtypes yielded additional prognostic information to the established clinicopathological variables and subclasses. For the HR+HER2- patients, the risk classification based on PAM50 ROR score was a strong independent prognostic factor. Among the HR+HER2- node negative patients receiving no adjuvant treatment (n=231), the PAM50 ROR score categorized 53.7% of the patients as low risk with

excellent prognosis (BrCa death 4.0%), 29.4% as intermediate risk (BrCa death 16.2%) and 16.9% as high risk (BrCa death 35.9%).

Among the patients who received adjuvant anti-estrogen treatment only (tamoxifen) (n=111), a low and similar risk of BrCa death was observed among the low and intermediate ROR risk groups. The high-risk group had a poor prognosis (BrCa death 33.3%, p<0.001), see Figure 1. The results show that the Prosigna test improves the classification of early breast cancer patients into prognostic groups, allowing for a more precise identification of future recurrence risk and improved basis for adjuvant treatment decisions.

According to the current treatment criteria for HR+HER2- breast cancer, the majority of patients receiving chemotherapy have a risk of future systemic disease of less than 20-25% with only small chance of chemotherapy benefit. Based on the results from analysis of 221 patients in the pre-application phase of EMIT, about 40-50% of the patients who would have received chemotherapy without use of Prosigna test, were classified as ROR low risk or luminal A ROR intermediate risk, corresponding to those patients who most likely would have little/no benefit from chemotherapy [72]. This is consistent with reports from the MINDACT trial that were recently published, showing that patients with clinical high risk and genomic low risk (by MammaPrint)(46% of the clinical high risk group) did not benefit from (or had minimal effect of) chemotherapy use[13]. However, the event rate was so low that it is difficult to draw firm conclusions from this study [13, 73]. But arguably, a large number of patients are over-treated with chemotherapy.

Only few studies have reported whether or not they included or excluded patients with lymph node micrometastases. In the TransATAC study [74] micrometastatic disease was not assessed and therefore treated as lymph node negative. In the MINDACT study [13], however, they were classified as lymph node positive. A variety of methods to detect and classify axillary micrometastasis exists and their prognostic significance remains unclear [75]. In clinical practice micrometastatic disease is classified as lymph node positive, but treated more similar to lymph node negative disease. An update of NICE guidelines (DG34 guidance [76]) published in 2018, therefore recommend that the Prosigna test should be available also for patients with lymph node micrometastases (pN1mi).

The feasibility of using Prosigna testing in routine diagnostics has also been assessed. Three pathologists from two hospitals (Oslo University hospital (OUS) and Vestre Viken (VV)) were trained in tissue selection for the test. For 20 consecutive patients enrolled in the Oslo2 study, the Prosigna test and the routine Ki67 test were performed in parallel. The study showed that the Prosigna result was available in time to be integrated into the final pathology report, or could be ready less than a week thereafter. Therefore, applying the test in routine diagnostics seems feasible and would not cause significant delay in treatment decisions. Based on all prevailing data, the Prosigna test has been through a mini-health technology assessment (mini- HTA) for use in HR+HER2-node negative patients.

Figure 1: Breast cancer specific survival (BCSS) and distant disease-free survival (DDFS) according to "Risk of Recurrence" score.

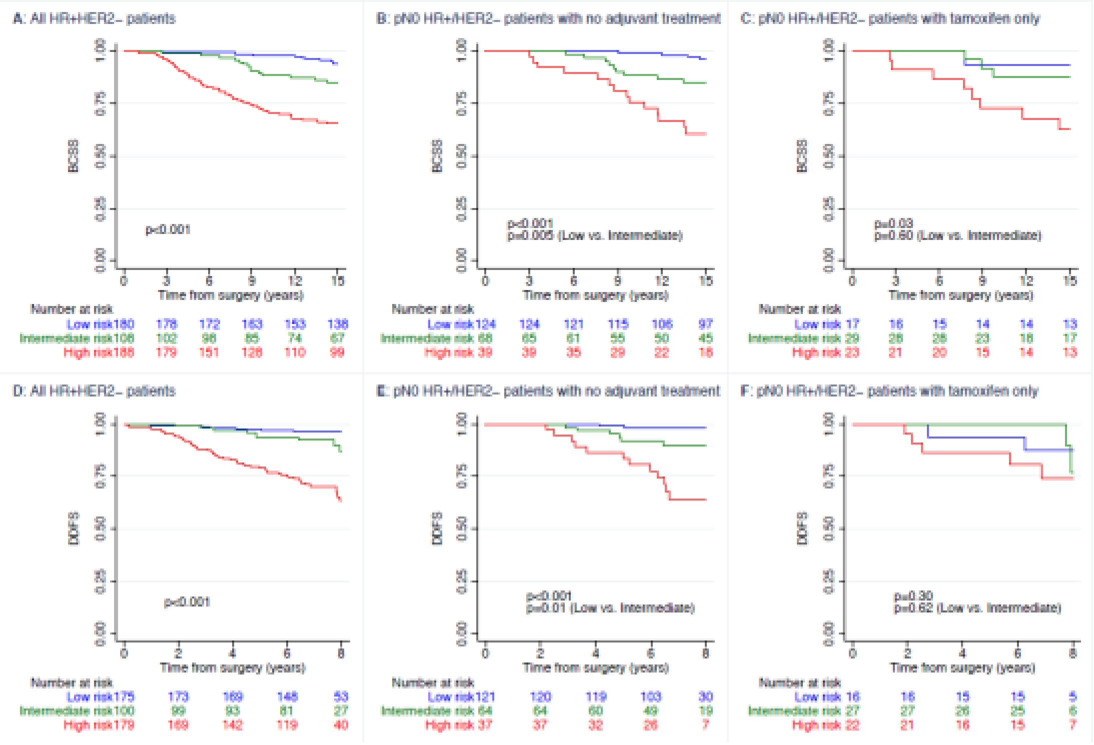

2.7 Resources and collaborative basis for the study

The research network behind this study constitutes highly skilled clinical and pathology groups, health economists, researchers from the National Advisory Unit on Late Effects after Cancer Treatment, statistician as well as translational and molecular profiling research groups including all health regions in Norway as presented in the organization overview below (Figure 2). A collaboration has been established with the cross-regional research network (funded by HSØ) that integrates clinical and basic/translational research and covers Oslo University Hospital, Akershus University Hospital, Vestre Viken Hospital, St Olav Hospital, Stavanger University Hospital, Kalnes Hospital, University of Oslo, Norwegian University of Science and Technology and further expanded to 18 hospitals in Norway. Thus, breast cancer centers in all parts of Norway participate. The project has also included close collaboration with C Caldas (Univ. of Cambridge, UK), C Perou (Lineberger CCC, Univ. of North Carolina, US) and A Prat (Vall d'Hebron Inst. of Oncology, Spain), all eminent researchers involved in the discovery, development and validation of molecular classifiers. Already there is an established network as a basis for the project, through OSBREACC (project number 2011042). This network has initiated several large observational studies (Oslo2) as well as intervention studies (NeoAVA, I-BCT), which includes tumor tissue sampling and collection of other biomaterial, whole genome microarray analyses, MR and MR related examinations both in vitro and in vivo. The core of the network was granted as a K.G. Jebsen Center for Breast Cancer in 2011, and thus has excellent basis for initiating this project. The study will also run in collaboration with the Norwegian Breast Cancer Group (NBCG), which opens for participation from breast cancer centers in all parts of Norway, as well as the Norwegian Cancer Registry.

Figure 2 Organization overview

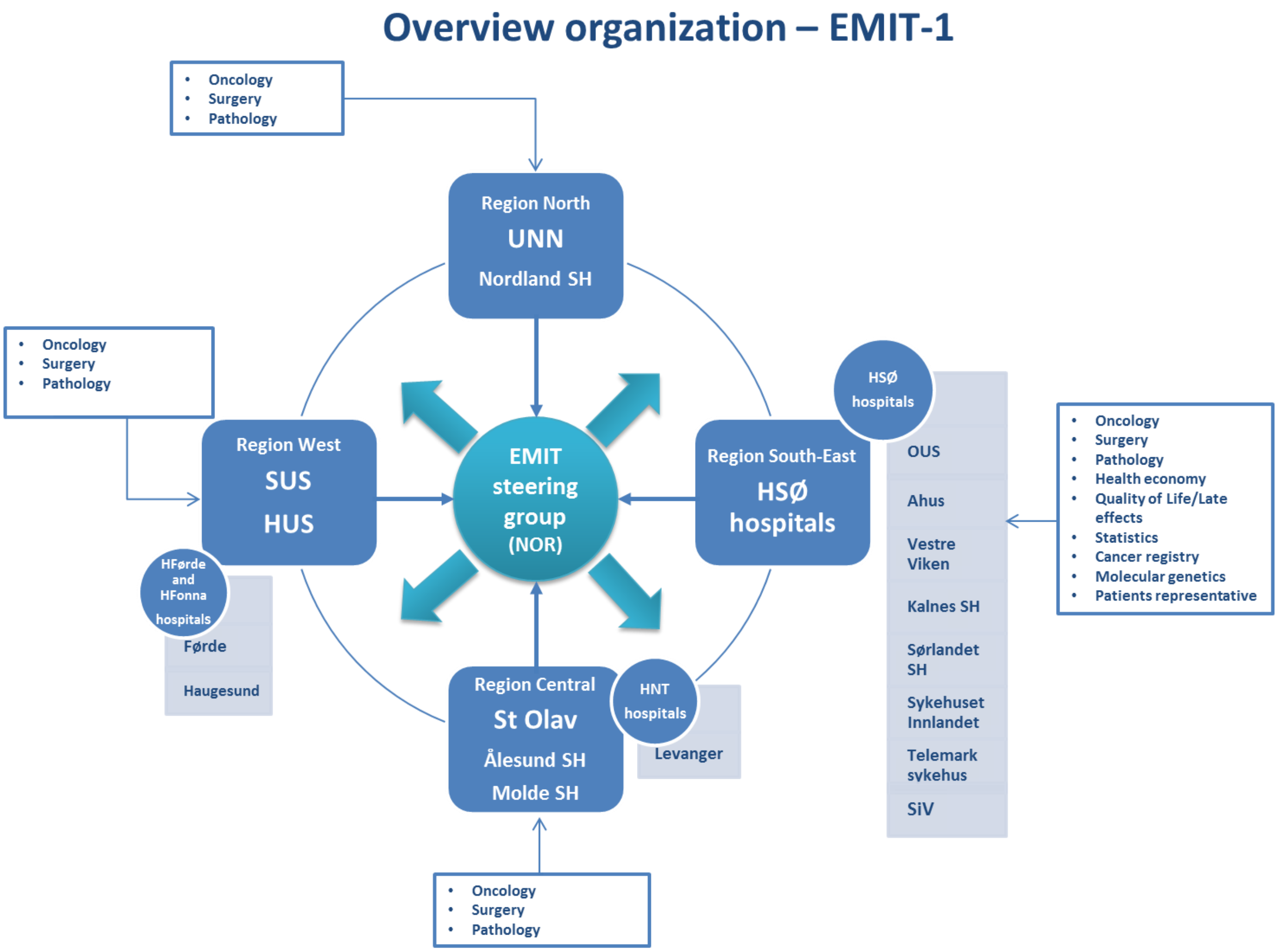

2.8 Rationale

The present project focuses on how to reduce both over- and undertreatment with adjuvant chemotherapy to a large number of breast cancer patients in Norway. A set of primary tumor prognostic factors can be analyzed for potential achievement of this. Furthermore, multi-parameter tests, including detailed molecular analysis of the primary tumors might further improve the selection of patients among the lymph node negative. The study seeks to advance the development of personalized treatment of patients with early breast cancer without lymph node metastasis, by the evaluation of multi-parameter analysis as a means of identifying those patients who are likely to benefit from chemotherapy whilst

sparing those who are unlikely to do so from an unnecessary and unpleasant treatment. In addition, the study seeks to gain experience from the use of the test also in patients with pT1 tumors having only micrometastatic spread to axillary lymph nodes.

3. OBJECTIVES OF THE STUDY

- To establish and optimize the use of molecular-based multi-parameter assay (i.e. Prosigna test) for adjuvant treatment decision in patients with hormone sensitive primary breast cancer without lymph node (macro)metastasis, compared to decisions based on standard practice. Differences in the number of patients receiving the various treatments using test-directed (Prosigna) treatment compared to standard adjuvant treatment recommendations will be registered.

- To determine recurrence-free interval (RFI) and distant recurrence-free interval (DRFI) after 5 years of follow-up (8 years from study start) for the lymph node negative patients.

- To identify differences in reported quality of life (QoL), late effects and working ability/capacity among patients receiving chemotherapy compared to those without chemotherapy.

- To establish the cost-effectiveness (i.e. health resource use in hospitals and society) of test-directed (Prosigna) treatment strategy compared to standard practice.

- To investigate the feasibility of the molecular classification-based treatment and achieve integrated knowledge on the consequences of nation-wide implementation of a molecular multi-parameter test.

4. STUDY DESIGN

Patients with node negative early breast cancer who have completed surgery and are classified as ER positive (HR positive) with ≥1% receptor expression are candidates for this study. In addition, patients with tumor size ≤ 20mm and lymph node micrometastases only may also be included. Patient can be

included after written informed consent has been obtained and eligibility has been established and approved. Overview of the study is illustrated in Figure 3. It will be organized as a multi-center study. The study will be run as a one-armed trial. Patients with appropriate primary tumor characteristics will be informed at first postoperative visit or after final conclusion from the postoperative multidisciplinary tumor board (MDT) meeting if postoperative visit is not part of the regular visits at the treating hospital. Potential study candidates may receive initial study information prior to the definitive selection of and information to the study candidates if this is the only realistic option (due to patient logistics) to ensure possibility for the patient to be included in the study (for example long travel distances).

Treatment recommendations will be based on the Prosigna test result, in addition to conventional clinicopathological parameters. The Prosigna test will be performed after study inclusion.

The study will recruit a total of 2150 lymph node negative patients, of whom approximately 1500 will not be recommended chemotherapy. After inclusion, the patients will be followed for breast cancer related events for at least 5 years.

Figure 3. Schematic overview of the study

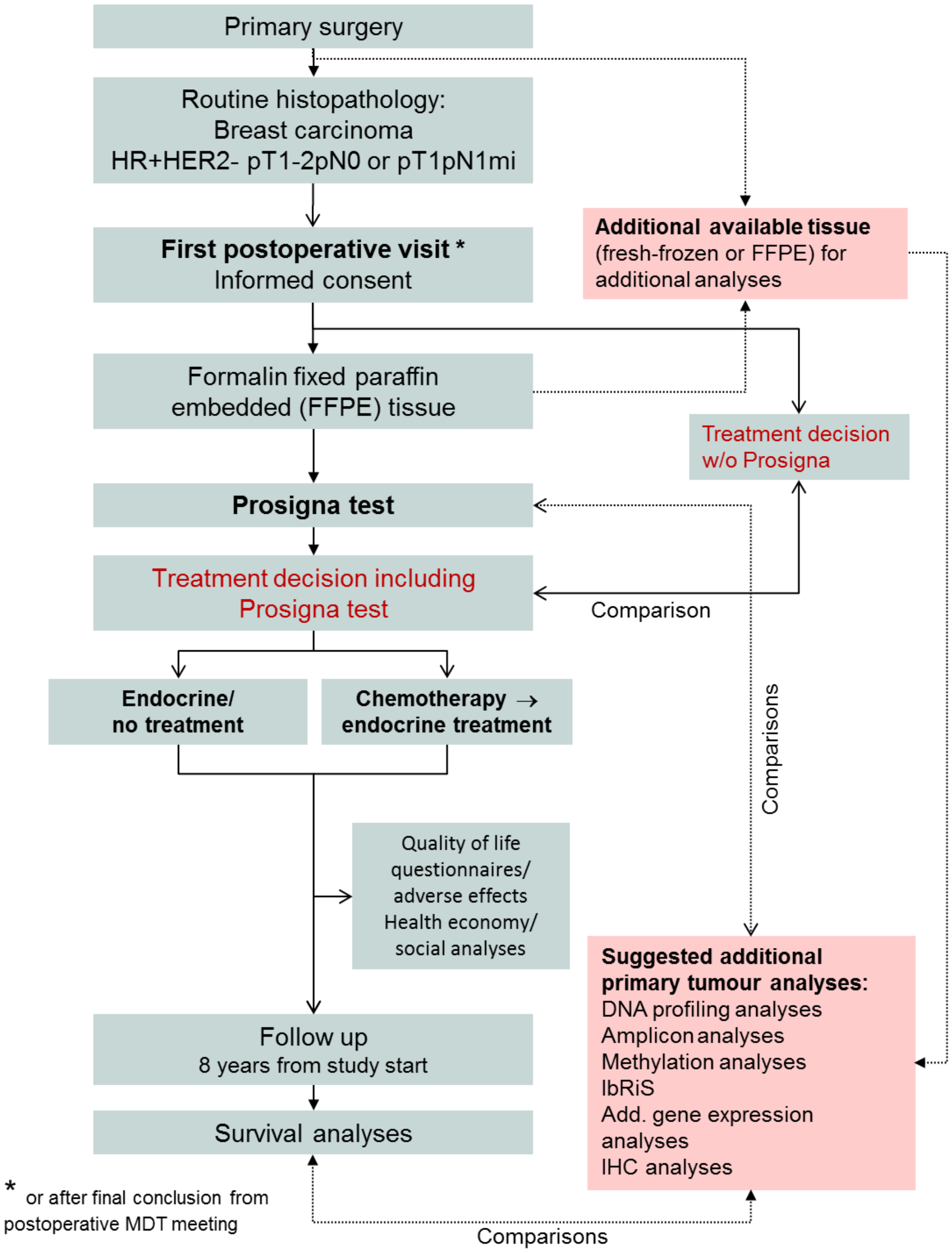

The observational Oslo2 study has included patients that may fulfill the criteria for inclusion into the current study. In that study, fresh tumor biopsies are collected at surgery and in-depth molecular analyses are performed (REK no: 538-07278a). The current study will be integrated with the Oslo2 study (in those hospitals participating in the Oslo2 study). In this respect, the tissue collected in the Oslo2 study will be used for the purpose of both the Oslo2 study, as well as the current study.

4.1 Surgery

The surgery will be performed according to standard recommendations and is a pre-study procedure. The surgical specimens are prepared according to routine (including FFPE), for final histopathological diagnosis. The surgical procedure and results should be documented in the Norwegian Breast Cancer Registry (NBCR, which will be the main Case Report Form (CRF) for the study). The study also opens for collection of fresh tumor specimens from patients participating in parallel studies. Such fresh tumor specimens will be prepared and stored at -80^{0}C for molecular analyses.

4.2 Scheduled visits/follow-up

During and after adjuvant treatment, the follow-up of patients will be according to usual care and NBCG guideline recommendations, including annual mammography and/or breast ultrasound. The events recorded during follow-up will be reported in the Norwegian Breast Cancer Registry (NBCR) which will be the main study CRF. The annual follow-up can be organized at the hospital or with the General Practitioner. At the minimum, relapse-free follow-up year 1, 2, 5 and at the end of study, or year 10 (what comes first), will be registered in NBCR. If recurrence occurs, this should usually be registered immediately after end of diagnostic work-up.

5. STUDY POPULATION

5.1 Target population

The study will include pN0/pN1mi patients with primary non-metastatic, hormone receptor positive, HER2 negative breast cancer as defined below.

5.2 Inclusion criteria

1. Written informed consent (informed consent document approved by the Independent Ethics Committee [IEC]) obtained prior to any study-specific procedure.
2. Female or male ≥ 18 years of age.
3. Able to comply with the protocol.
4. Primary surgery completed or if re-resection needed, a change from pT1 to pT2 categorization is not expected.
5. Histologically confirmed adenocarcinoma of the breast ≤ 5.0 cm in size (pT1-2) without metastasis to regional lymph nodes (pN0) or ≤ 20 mm in size (pT1) with lymph node micrometastases only (pN1mi).
6. Primary tumor concluded as ER positive (≥ 1% expression) HER2 negative.

5.3 Exclusion criteria

1. Metastasis to regional lymph nodes or distant sites/organs. Lymph node micrometastases (pN1mi) is allowed if pT1 (confer subsection 5 above).
2. Previous treatment for localized breast cancer. Previous treatment for DCIS is allowed.
3. HER2 positive.
4. ER negative (<1% expression).
5. Other concomitant or earlier carcinoma less than five years prior to the breast cancer diagnosis, except for basal cell carcinoma and *in situ* cervix cancer.
6. Use of or participation in intervention trials testing treatment with any investigational anti-cancer drug. Participation in other types of intervention trials is allowed (such participation needs to be registered).
7. Evidence of any other disease or condition that by the investigator is considered to impede follow-up of the patients.

6. SCHEDULE OF ASSESSMENTS, PROCEDURES AND FOLLOW-UP

Table 1 Study plan

Time	Pre incl[a]	D1[g]	3 months from trial entry	6 months from trial entry	Y1	Y2	Y3	Y4	Y5	Y6	Y7	Y8	Y9	Y10
Signed informed consent		✓												
Metastasis screening[b]	(✓)				(✓)	(✓)	(✓)	(✓)	(✓)	(✓)	(✓)	(✓)	(✓)	(✓)
Histopathological tumor assessment[c]	✓													
Prosigna test[d]		✓[d]												
Functional status[e]	✓				✓	✓	✓	✓	✓	✓	✓	✓	✓	✓
Patient-reported assessments (QLQ)		✓[k]	✓[l]	✓[l]	✓[l]	✓[l]			✓[l]					
Mammography/breast ultrasound					✓[i]	✓[i]	✓[i]	✓[i]	✓[i]	✓[i]	✓[i]	✓[i]	✓[i]	✓[i]
Clinical examination[f]	✓	✓[h]			✓	✓	✓	✓	✓	✓	✓	✓	✓	✓
Primary tumor characterization[j]		✓ (D1 to Y5)												

Footnotes:

a. If not otherwise stated, the assessments must take place within 45 days before inclusion.
b. Only necessary if clinically indicated. Metastasis screening may include bone scan (or MRI) and chest x-ray/US liver or CT thorax/abdomen)
c. Standard routine histopathology (including HER2, Ki67, ER/PgR status and Grade).
d. Molecular-based multi-parameter test (Prosigna) on FFPE tumor material. Assessments should be performed within 21 days after inclusion.
e. Functional status (work ability) at follow-up according to NBCR.
f. Locoregional examination of breast/chest wall and regional lymph nodes, additional examination if clinically indicated
g. D1= at inclusion.
h. Only needed if clinically indicated (changes in clinical status from pre-inclusion)
i. Patients will be followed according to NBCG recommendations including annual visits (either at the hospital or by the General Practitioner (GP), with the first visit approximately 12 months from preoperative mammography, the following visits should be performed at 12 months interval (+/- 2 months). At follow-up, the need of ultrasound is usually decided by the radiologist.
j. The various molecular analyses of the primary tumor will be performed at different time points during and after the study, but most of the analyses will be completed within the first 5 years.
k. Assessments should be performed within 20 days post-inclusion. Reminders will be scheduled according to the eCRF Viedoc User Acceptance Test Document V1.0 , 12.04.2018
l. Assessments should be performed within 60 days. Reminders will be scheduled according to the eCRF Viedoc User Acceptance Test Document V1.0, 12.04.2018

6.1 Screening

The primary tumor analysis of HER2, Ki67, ER/PgR, Grade, pT and and pN status are performed on a routine basis in accordance with the national guidelines. These analyses identify candidates for study inclusion, and should be performed before informed consent is obtained. The Prosigna test will be

performed after informed consent, in accordance with the instructions in the protocol for assessment of the molecular-based multi-parameter test.

6.2 Procedure and follow-up plan after inclusion to the study

Included patients will receive systemic adjuvant treatment according to the molecular classification algorithm (Prosigna) in concert with routine histopathology, in line with the principles for adjuvant systemic treatment decisions in Norway (www.nbcg.no). Radiation treatment will be given according to what is recommended. The patients will be followed annually, either at the local hospital/study center or with the GP. At the minimum, relapse-free follow-up year 1, 2, 5 and at the end of study, or year 10 (what comes first), will be registered in NBCR. If recurrence occurs, this should usually be registered immediately after end of diagnostic work-up.

6.3 Study assessments

At inclusion and follow-up visits

- Signed informed consent (at inclusion).
- Demographics (age, sex) (at inclusion), medical non-breast cancer related history (not to be included in CRF, only in the medical record).
- Physical examination including locoregional examination of breast/chest wall and regional lymph nodes, additional examination if clinically indicated.
- Functional status (work ability) according to NBCR.
- Mammography and if indicated breast ultrasound, if not bilateral mastectomy, before surgery and at follow-up visits.
- Relapse status (at follow-up).

In addition to information from the patient questionnaires, patient medication (including continuation of endocrine treatment or other breast cancer treatment related medication) will be collected through the Norwegian Prescription Database, and occupational disability/sick leave through Statistics Norway (SSB). As the recorded patients' data in the project will be stored for a very long time, it will later be relevant to link information with information from the Cancer Registry of Norway and the Death Cause Registry.

6.4 Patient related procedures

6.4.1 Tumor tissue sampling

This study opens for analysis of collected fresh tumor specimens from patients participating in additional studies, if specific approval has been obtained from the actual study/studies.

6.4.2 Blood sampling

If decided, peripheral blood will be drawn in EDTA tubes for the collection of plasma and buffy coat. The plasma and buffy coat will be separated and stored frozen at -80^{0}C for later analysis. These blood samples, as well as other potentially available collected blood samples, may be used for explorative analyses in the current study.

6.4.3 Patient reported assessments

At baseline, 3 months, 6 months, 1 year, 2 years and at 5 year follow-up all patients will be asked to answer questionnaires consisting of established scales with good psychometric properties and single items covering socio-demographics, comorbidity, work ability, general health and quality of life (including RAND36 and the EQ-5D), fatigue (fatigue questionnaire[77]), anxiety (GAD-7 questionnaire) and depressive symptoms [78], life style, sleep and breast cancer specific symptoms and complaints (EORTC QLQ-BR23, FACT-B and FACT-ES). The main quality of life outcome in the present study will be level of fatigue and chronic fatigue (CF) measured at the time of the cancer diagnosis and at 5 years follow-up with both somatic and psychological factors as explanatory variables assessed at the same time points.

6.4.4 Health economic assessments

Formal economic evaluation of cost-benefit will be performed based on simulation of treatment paths. First, an analysis of the expected cost of the interventions (patient and hospital costs) will be performed. The second step will involve an estimation of the benefits. A likely key benefit is the potential reduction in health resource use (for instance late effects that need health service and sick-leave) resulting from fewer patients treated with chemotherapy. The size of the cost/benefits will be estimated from data provided by the questionnaires as mentioned above (section 6.4.3) as well as from other external sources (section 6.3).

6.5 Primary tumor analyses

Except for a small part of tumor being fresh frozen if the patient participates in a parallel study that collects fresh tumor tissue, the entire primary tumor (but with a maximum of 5 paraffin blocks) will be embedded in paraffin for subsequent analysis.

Standard analysis of the primary tumor in the breast will be performed as part of the routine diagnostics at the patient's local hospital. According to national guidelines this will include the following: localization in the breast, tumor size, histological type and tumor grade, mitotic count, amount of ductal carcinoma in situ (DCIS) within and outside of the tumor, together with distance from infiltrating tumor and distance from DCIS, to nearest surgical resection border.

The number of lymph nodes removed should be stated. All included patients will be lymph node negative (pN0 including (i+) and (mol+)) or lymph node positive with tumor deposits ≤2 mm (pN1mi) and tumor size ≤ 20mm, according to current WHO staging.

6.5.1 Analysis of HER2, ER/PgR, Ki67 and Grade

HER2 analysis will be performed at the local hospital or referred to a central laboratory, depending on local routines. The HER2 status may be assessed by immunohistochemistry, in situ hybridization or both, in accordance with the prevailing national guidelines announced by the breast pathology group in NBCG.

Immunohistochemical staining for the expression of estrogen- and progesterone receptors will also be performed locally as part of primary routine diagnostics, on the condition that staining protocols are validated by an external quality assurance program and follow current national/international guidelines (from NBCG/ASCO/CAP). The pathology report should state the proportion of estrogen- and/or progesterone receptor positive cells.

Ki67 analysis will also be performed locally, and according to the prevailing recommendations distributed by the pathology group in NBCG. Briefly, a tumor block from the surgery specimen, containing the area of highest tumor cell cellularity and tumor grade, also including the tumor periphery, is chosen for analysis. The area of highest tumor cell proliferation is selected and marked. Within the selected area 500 tumor cell nuclei are evaluated by help of a raster, and the result, as the percentage of Ki67-positive nuclei is calculated.

Tumor grading will be performed according to Elston and Ellis [79], and as further specified in criteria distributed nationally by the breast pathology group of NBCG. Ductal carcinoma in situ will be graded according to the guidelines from the latter, following Van Nuys grading.

6.5.2 Molecular-based multi-parameter analysis, Prosigna™

The PAM50 ROR analysis is now available as a standardized FDA approved, CE marked assay termed Prosigna™ using digital bar code technology (NanoString Technologies Inc., subsequently transferred to Veracyte Inc). Among the routine pathology, one FFPE tissue block representative for the primary tumor and usually containing area of highest tumor grade, will be selected by a dedicated pathologist. The sectioning for the molecular-based multi-parameter test will be performed after completion of sectioning for the standard routine diagnostic analysis (which includes sectioning for hematoxylin staining, estrogen- and progesterone receptor, Ki67 and HER2, see section 6.5.1). The tumor area on the sections will be identified by a pathologist, and then micro dissected and RNA-isolated according to the Prosigna procedure. Using the NanoString nCounter system, the ROR score will be provided using the NanoString protocol (www.prosigna.com).

6.5.3 Other molecular analyses of tumor tissue

FFPE tumor tissue (and potentially fresh frozen) from the patients included in the current study will be available for further translational research (besides Prosigna™ analysis, see section 6.5.2). The intended analyses aim to achieve knowledge to refine future tumor classification and identification of new tumor markers relevant for clinical use. Already planned studies include prospective validation of IC-10 DNA copy number analyses, DNA methylation analysis of 40 selected genes, integration of genomic and transcriptomic data, analyses of immune-related profiles and other gene expression patterns, Ki67, adjusted mitotic score, centrosome amplification and specific microRNA-analyses. In addition, more explorative projects will be designed based on sequencing of tumors from patients included in current study, the Oslo2 study (RNA/DNA from fresh primary tumor tissue) and other studies consented by the same patients.

7. STATISTICAL CONSIDERATIONS AND ANALYTICAL PLAN

7.1 Determination of patient number / sample size

The study will report recurrence-free interval (RFI) and distant recurrence-free interval (DFRI) after 5 years follow-up (approximately 8 years from study start) for the lymph node negative population, the subgroup where use of gene expression profiling is unanimously included in International Guidelines to guide chemotherapy treatment decisions. In the retrospective EMIT0 study, including an equivalent study population, but with less use of adjuvant systemic treatment than currently recommended, the breast cancer specific death at 15 years follow-up was 4% for patients with a low risk ROR score (Ohnstad et al, BCR, 2017). Furthermore, at 5 years follow up, 3.9% (10/257) of patients with Luminal A and Luminal B subtype with a low or intermediate ROR score who received either no systemic adjuvant treatment (n=209) or endocrine treatment only (n=48) experienced systemic recurrence. Following the anticipated reduced and altered use of adjuvant chemotherapy by inclusion of Prosigna in today's decision-making, the current project aims to retain excellent survival for the patient group not undergoing treatment with chemotherapy, a group expected to increase in size compared to decisions based upon conventional histopathology assessments. With inclusion of close to 2150 lymph node negative patients during a period of approximately 3-3.5 years,, it is estimated that at least 1500 patients will not be recommended chemotherapy. This is based on both estimates of current treatment allocation without Prosigna and conservative estimates of changes in risk classification by use of the Prosigna test (Ohnstad et al, BCR, 2017). If the distant recurrence-free interval is 96%, statistical analysis reveals a 95% confidence interval for distant recurrence-free interval of 95-97% is obtained with this sample size of chemotherapy untreated patients.

The benefit of chemotherapy is mainly restricted to the first 5 years, and the maximum absolute benefit from chemotherapy would be less than 1.5% (with DRFI around 96%) if the proportional

benefits of chemotherapy (HR 0.7) apply uniformly to all patients irrespective of tumor characteristics assessed by conventional histopathology [80]. This emphasizes very small potential benefit for those patients selected to no adjuvant chemotherapy, which needs to be offset against the toxicity experienced by all patients treated with chemotherapy.

The primary tumor molecular analyses will be performed within a period of 3-5 years from the start of the project. The primary analyses are planned 8 years after study start, but further explorative analyses of clinical outcome are planned after 10 years follow up of the patients.

The sample size of the subgroup of patients with lymph node micrometastases and T≤ 20mm is expected to be small. As this group is categorized as lymph node positive according to the TNM classification and may have an inferior prognosis compared to the lymph node negative patients, this subgroup will not be included in the survival analyses and consequently not included in the sample size calculation above. However, this subgroup will be included in descriptive statistics, analysis of quality of life and health care costs. Further explorative analyses will be performed for this subgroup separately.

7.2 Statistical analysis of study data

The following statistical analyses will be performed

1. Differences in the number of patients receiving the various treatments using the Prosigna test compared to adjuvant treatment recommendations without Prosigna
2. Determine DRFI after 5 years of follow-up (8 years from study start) for the lymph node negative population
3. Determine RFI after 5 years of follow-up (8 years from study start) for the lymph node negative population
4. Differences in reported quality of life (QoL), late effects and working ability/capacity among patients receiving chemotherapy compared to those who did not receive chemotherapy
5. Health care costs and benefit for the hospitals and society using the Prosigna test, compared to the costs estimated when based on adjuvant treatment recommendations without Prosigna

Available data include histopathological data, risk of recurrence score, treatment decision, clinical outcome (relapse or death due to breast cancer), quality of life outcomes such as level of fatigue and chronic fatigue (CF) (with both somatic and psychological factors as explanatory variables) as well as work ability and health resource use. Furthermore, external information such as sick leaves will be collected.

To identify factors associated with survival, methods to be applied include cumulative incidence and Kaplan-Meier plots as well as Cox regression analyses. To statistically assess the association between breast cancer subgroups found in the analysis described above and clinical endpoints, statistical tests for comparison of survival in two or more groups (log rank test) will be used. Multivariate regression analysis will be used to adjust for other factors (such as age). Possible violations of the proportional

hazards assumption inherent in both types of tests above will be detected using graphical methods as well as formal tests based on the Schoenfeld residuals. Other statistical methods of analysis can also be considered.

Standard descriptive statistics and multiple linear regressions will be employed in the analyses of continuous responses (e.g. level of fatigue). Prognostic factors of categorical responses (e.g. treatment decision and chronic fatigue) will be analyzed by multiple logistic regressions.

Formal economic evaluation of cost-benefit will be performed based on simulation of treatment paths. Firstly, an analysis of the expected cost of the interventions (patient and hospital costs) will be performed. The second step will involve an estimation of the benefits. The size of the benefits will be estimated from data provided by the clinical study as well as from other external sources. Finally, the information on costs and benefits will be used in a formal model that simulates the various treatment paths a patient can take (with the associated probabilities, costs and benefits). These simulations will lead to conclusion about the cost per quality adjusted life of the new procedures compared to the current system as well as more general social gains (including benefits such as reduced sick pay).

8. ETHICS AND GENERAL STUDY ADMINISTRATION

8.1 Ethics

The study will be performed in accordance with the Declaration of Helsinki and Norwegian law. The study will only start after approval from the Regional Ethics Committee. All patients will be given study specific identification codes and all data will be stored and handled in a secure database.

It is the responsibility of the investigator, or a person designated by the investigator, to obtain written informed consent from each patient participating in the study, after adequate explanation of the aims, methods, anticipated benefits and potential hazards of the study. The patients are completely free to withdraw from the study at any time for any reason.

8.2 Study organization

Per 23.10.17, the following Norwegian hospitals support and intend to participate in the study (see Figure 2):

<u>Region South East:</u>

Oslo University Hospital

Akershus University Hospital

Østfold Hospital, Kalnes

Sørlandet Hospital

Vestre Viken Hospital

Innlandet Hospital

Vestfold Hospital

Telemark Hospital

<u>Region West:</u>

Haukeland University Hospital

Stavanger University Hospital

<u>Region Central:</u>

St. Olavs Hospital

Molde Hospital

Ålesund Hospital

<u>Region North:</u>

University Hospital of North Norway

Nordland Hospital

Principle investigator:

Bjørn Naume, Oslo University Hospital

See appendix 1 for updated list of participants.

8.3 Monitoring

It is aimed at monitoring clinical patient data from at least 10% of the included patients. The monitoring plan will be described in a separate document. During inclusion, the coordinating center will ensure that updated versions of all relevant study documents are on-site and monitor the compliance to the protocol and study specific procedures by regular collection of the inclusion forms and delegation-logs. If necessary, on-site monitoring visits will be performed. Thereafter, it is

considered sufficient to receive the NBCR follow-up reports for the patients (function as Case Report Forms). At the end of scheduled follow-up, the recorded (in NBCR) clinical status for a subset of patients (relapse-free, local relapse, distant relapse) will be controlled by direct request for these parameters from the hospitals journal. The monitor will have access to all necessary records of the trial needed to verify the entries on the Case Report Form provided that patient confidentiality is maintained. The verification of the Case Report Form data must be by direct inspection of source documents.

8.4 Case Report Form and Data management

For each patient enrolled, an inclusion-to-study CRF, also including “pre-Prosigna-test” suggested decision of adjuvant systemic treatment must be completed and signed by the Trial investigator or authorized delegate from the study staff. All following patient data will be registered directly in the NBCR database (or Medinsight). Each site will have their own site number and all patients will be allocated a patient number. The Investigator is responsible for ensuring the accuracy, completeness, legibility and timeliness of the data recorded. All analyses of patient data will be performed “pseudonymized”, only recognized with the patient number. The link between the patient identity and the patient number will be securely stored in a password-protected inclusion-to-study database at the Oslo University Hospital’s secured server, separate from other patient data.

Results from primary tumor molecular analyses will be stored “pseudonymized” in a study database at Oslo University Hospital, linked to the clinical patient data by the patient number.

8.5 Archiving

The investigator must maintain adequate and accurate records to enable the conduct of the study to be fully documented and the study data to be subsequently verified. All study documents, Investigator’s Study File and patient clinical source documents, should be kept on file for at least 15 years after the final study report is available according to Norwegian law.

8.6 Inspections

The study may be inspected by Norwegian or international Health Authorities. The investigator should make all study documents including patient source documents available for the inspectors after appropriate notification.

8.7 Confidentiality of trial documents and subject records

The investigator must assure that patients' anonymity will be maintained. On CRFs or other documents submitted to unauthorized parties, patients should be identified by an identification code. The investigator must keep a patient enrolment and identification log showing codes, names and addresses. This log and patients' written consent forms should be maintained in strict confidence. All personal information we collect for this study is strictly confidential and will be stored and handled according to the EU General Data Protection Regulation as of May 2018.

8.8 Publication

The protocol, the report and any document relating thereto including the results of this clinical study is the exclusive property of the Steering Committee (SC). The results of this study will be published in international medical journals, and will also be communicated to the general population whenever appropriate. In accordance with standard editorial and ethical practice, the data will not be published as individual center data. All manuscripts will be written by the Steering committee or persons to whom delegated this responsibility from the SC. The SC must approve all publications.

9. REFERENCES

1. Carter CL, Allen C, Henson DE: **Relation of tumor size, lymph node status, and survival in 24,740 breast cancer cases**. *Cancer* 1989, **63**(1):181-187.
2. Foulkes WD, Reis-Filho JS, Narod SA: **Tumor size and survival in breast cancer--a reappraisal**. *Nature reviews Clinical oncology* 2010, **7**(6):348-353.
3. Christiansen P, Bjerre K, Ejlertsen B, Jensen MB, Rasmussen BB, Laenkholm AV, Kroman N, Ewertz M, Offersen B, Toftdahl DB *et al*: **Mortality rates among early-stage hormone receptor-positive breast cancer patients: a population-based cohort study in Denmark**. *Journal of the National Cancer Institute* 2011, **103**(18):1363-1372.
4. Goldhirsch A, Winer EP, Coates AS, Gelber RD, Piccart-Gebhart M, Thurlimann B, Senn HJ: **Personalizing the treatment of women with early breast cancer: highlights of the St Gallen International Expert Consensus on the Primary Therapy of Early Breast Cancer 2013**. *Annals of oncology : official journal of the European Society for Medical Oncology* 2013, **24**(9):2206-2223.
5. Goldhirsch A, Wood WC, Gelber RD, Coates AS, Thurlimann B, Senn HJ: **Progress and promise: highlights of the international expert consensus on the primary therapy of early breast cancer 2007**. *Annals of oncology : official journal of the European Society for Medical Oncology* 2007, **18**(7):1133-1144.

6. Lee AH, Ellis IO: **The Nottingham prognostic index for invasive carcinoma of the breast**. *Pathology oncology research : POR* 2008, **14**(2):113-115.
7. Blows FM, Driver KE, Schmidt MK, Broeks A, van Leeuwen FE, Wesseling J, Cheang MC, Gelmon K, Nielsen TO, Blomqvist C *et al*: **Subtyping of Breast Cancer by Immunohistochemistry to Investigate a Relationship between Subtype and Short and Long Term Survival: A Collaborative Analysis of Data for 10,159 Cases from 12 Studies**. *PLOS Medicine* 2010, **7**(5):e1000279.
8. Geradts J, Bean SM, Bentley RC, Barry WT: **The oncotype DX recurrence score is correlated with a composite index including routinely reported pathobiologic features**. *Cancer investigation* 2010, **28**(9):969-977.
9. Synnestvedt M, Borgen E, Russnes HG, Kumar NT, Schlichting E, Giercksky KE, Karesen R, Nesland JM, Naume B: **Combined analysis of vascular invasion, grade, HER2 and Ki67 expression identifies early breast cancer patients with questionable benefit of systemic adjuvant therapy**. *Acta oncologica (Stockholm, Sweden)* 2013, **52**(1):91-101.
10. Strand C, Ahlin C, Bendahl PO, Fjallskog ML, Hedenfalk I, Malmstrom P, Ferno M: **Combination of the proliferation marker cyclin A, histological grade, and estrogen receptor status in a new variable with high prognostic impact in breast cancer**. *Breast cancer research and treatment* 2012, **131**(1):33-40.
11. Iqbal J, Ginsburg OM, Wijeratne TD, Howell A, Evans G, Sestak I, Narod SA: **Endometrial cancer and venous thromboembolism in women under age 50 who take tamoxifen for prevention of breast cancer: a systematic review**. *Cancer treatment reviews* 2012, **38**(4):318-328.
12. Zambetti M, Moliterni A, Materazzo C, Stefanelli M, Cipriani S, Valagussa P, Bonadonna G, Gianni L: **Long-term cardiac sequelae in operable breast cancer patients given adjuvant chemotherapy with or without doxorubicin and breast irradiation**. *Journal of clinical oncology : official journal of the American Society of Clinical Oncology* 2001, **19**(1):37-43.
13. Cardoso F, van't Veer LJ, Bogaerts J, Slaets L, Viale G, Delaloge S, Pierga JY, Brain E, Causeret S, DeLorenzi M *et al*: **70-Gene Signature as an Aid to Treatment Decisions in Early-Stage Breast Cancer**. *The New England journal of medicine* 2016, **375**(8):717-729.
14. Cuzick J, Dowsett M, Pineda S, Wale C, Salter J, Quinn E, Zabaglo L, Mallon E, Green AR, Ellis IO *et al*: **Prognostic value of a combined estrogen receptor, progesterone receptor, Ki-67, and human epidermal growth factor receptor 2 immunohistochemical score and comparison with the Genomic Health recurrence score in early breast cancer**. *Journal of clinical oncology : official journal of the American Society of Clinical Oncology* 2011, **29**(32):4273-4278.
15. Sestak I, Buus R, Cuzick J, Dubsky P, Kronewett R, Ferree S, Sgroi D, Schnabel C, Baehner R, Mallon E *et al*: **Comprehensive comparison of prognostic signatures for breast cancer in TransATAC**. In: *SABCS 2016.* San Antonio; 2016.
16. Filipits M, Rudas M, Jakesz R, Dubsky P, Fitzal F, Singer CF, Dietze O, Greil R, Jelen A, Sevelda P *et al*: **A new molecular predictor of distant recurrence in ER-positive, HER2-negative breast cancer adds independent information to**

conventional clinical risk factors. *Clinical cancer research : an official journal of the American Association for Cancer Research* 2011, **17**(18):6012-6020.
17. Dowsett M, Sestak I, Lopez-Knowles E, Sidhu K, Dunbier AK, Cowens JW, Ferree S, Storhoff J, Schaper C, Cuzick J: **Comparison of PAM50 risk of recurrence score with oncotype DX and IHC4 for predicting risk of distant recurrence after endocrine therapy**. *Journal of clinical oncology : official journal of the American Society of Clinical Oncology* 2013, **31**(22):2783-2790.
18. Cheang MC, Voduc D, Bajdik C, Leung S, McKinney S, Chia SK, Perou CM, Nielsen TO: **Basal-like breast cancer defined by five biomarkers has superior prognostic value than triple-negative phenotype**. *Clinical cancer research : an official journal of the American Association for Cancer Research* 2008, **14**(5):1368-1376.
19. Goldhirsch A, Glick JH, Gelber RD, Coates AS, Thürlimann B, Senn HJ: **Meeting Highlights: International Expert Consensus on the Primary Therapy of Early Breast Cancer 2005**. *Annals of Oncology* 2005, **16**(10):1569-1583.
20. Goldhirsch A, Ingle JN, Gelber RD, Coates AS, Thurlimann B, Senn HJ: **Thresholds for therapies: highlights of the St Gallen International Expert Consensus on the primary therapy of early breast cancer 2009**. *Annals of oncology : official journal of the European Society for Medical Oncology* 2009, **20**(8):1319-1329.
21. **https://www.kreftregisteret.no/Generelt/Publikasjoner/Arsrapport-fra-kvalitetsregistrene/Arsrapport-for-brystkreft/**
22. Sotiriou C, Wirapati P, Loi S, Harris A, Fox S, Smeds J, Nordgren H, Farmer P, Praz V, Haibe-Kains B *et al*: **Gene expression profiling in breast cancer: understanding the molecular basis of histologic grade to improve prognosis**. *Journal of the National Cancer Institute* 2006, **98**(4):262-272.
23. Metzger Filho O, Ignatiadis M, Sotiriou C: **Genomic Grade Index: An important tool for assessing breast cancer tumor grade and prognosis**. *Critical reviews in oncology/hematology* 2011, **77**(1):20-29.
24. Harbeck N, Thomssen C: **A new look at node-negative breast cancer**. *The oncologist* 2011, **16 Suppl 1**:51-60.
25. de Azambuja E, Cardoso F, de Castro G, Jr., Colozza M, Mano MS, Durbecq V, Sotiriou C, Larsimont D, Piccart-Gebhart MJ, Paesmans M: **Ki-67 as prognostic marker in early breast cancer: a meta-analysis of published studies involving 12,155 patients**. *British journal of cancer* 2007, **96**(10):1504-1513.
26. Stuart-Harris R, Caldas C, Pinder SE, Pharoah P: **Proliferation markers and survival in early breast cancer: a systematic review and meta-analysis of 85 studies in 32,825 patients**. *Breast (Edinburgh, Scotland)* 2008, **17**(4):323-334.
27. Bevilacqua P, Verderio P, Barbareschi M, Bonoldi E, Boracchi P, Dalla Palma P, Gasparini G: **Lack of prognostic significance of the monoclonal antibody Ki-S1, a novel marker of proliferative activity, in node-negative breast carcinoma**. *Breast cancer research and treatment* 1996, **37**(2):123-133.
28. Jung SY, Han W, Lee JW, Ko E, Kim E, Yu JH, Moon HG, Park IA, Oh DY, Im SA *et al*: **Ki-67 expression gives additional prognostic information on St. Gallen**

2007 and Adjuvant! Online risk categories in early breast cancer. *Annals of surgical oncology* 2009, **16**(5):1112-1121.

29. Clahsen PC, van de Velde CJ, Duval C, Pallud C, Mandard AM, Delobelle-Deroide A, van den Broek L, van de Vijver MJ: **The utility of mitotic index, oestrogen receptor and Ki-67 measurements in the creation of novel prognostic indices for node-negative breast cancer**. *European journal of surgical oncology : the journal of the European Society of Surgical Oncology and the British Association of Surgical Oncology* 1999, **25**(4):356-363.
30. Joensuu H, Isola J, Lundin M, Salminen T, Holli K, Kataja V, Pylkkanen L, Turpeenniemi-Hujanen T, von Smitten K, Lundin J: **Amplification of erbB2 and erbB2 expression are superior to estrogen receptor status as risk factors for distant recurrence in pT1N0M0 breast cancer: a nationwide population-based study**. *Clinical cancer research : an official journal of the American Association for Cancer Research* 2003, **9**(3):923-930.
31. Dowsett M, Nielsen TO, A'Hern R, Bartlett J, Coombes RC, Cuzick J, Ellis M, Henry NL, Hugh JC, Lively T *et al*: **Assessment of Ki67 in breast cancer: recommendations from the International Ki67 in Breast Cancer working group**. *Journal of the National Cancer Institute* 2011, **103**(22):1656-1664.
32. Goldhirsch A, Wood WC, Coates AS, Gelber RD, Thurlimann B, Senn HJ: **Strategies for subtypes--dealing with the diversity of breast cancer: highlights of the St. Gallen International Expert Consensus on the Primary Therapy of Early Breast Cancer 2011**. *Annals of oncology : official journal of the European Society for Medical Oncology* 2011, **22**(8):1736-1747.
33. Cheang MC, Chia SK, Voduc D, Gao D, Leung S, Snider J, Watson M, Davies S, Bernard PS, Parker JS *et al*: **Ki67 index, HER2 status, and prognosis of patients with luminal B breast cancer**. *Journal of the National Cancer Institute* 2009, **101**(10):736-750.
34. Polley MY, Leung SC, Gao D, Mastropasqua MG, Zabaglo LA, Bartlett JM, McShane LM, Enos RA, Badve SS, Bane AL *et al*: **An international study to increase concordance in Ki67 scoring**. *Modern pathology : an official journal of the United States and Canadian Academy of Pathology, Inc* 2015, **28**(6):778-786.
35. Coates AS, Winer EP, Goldhirsch A, Gelber RD, Gnant M, Piccart-Gebhart M, Thurlimann B, Senn HJ: **Tailoring therapies--improving the management of early breast cancer: St Gallen International Expert Consensus on the Primary Therapy of Early Breast Cancer 2015**. *Annals of oncology : official journal of the European Society for Medical Oncology* 2015, **26**(8):1533-1546.
36. Harris LN, Ismaila N, McShane LM, Andre F, Collyar DE, Gonzalez-Angulo AM, Hammond EH, Kuderer NM, Liu MC, Mennel RG *et al*: **Use of Biomarkers to Guide Decisions on Adjuvant Systemic Therapy for Women With Early-Stage Invasive Breast Cancer: American Society of Clinical Oncology Clinical Practice Guideline**. *Journal of clinical oncology : official journal of the American Society of Clinical Oncology* 2016, **34**(10):1134-1150.

37. **Effects of chemotherapy and hormonal therapy for early breast cancer on recurrence and 15-year survival: an overview of the randomised trials**. *Lancet (London, England)* 2005, **365**(9472):1687-1717.
38. Francis PA, Regan MM, Fleming GF, Lang I, Ciruelos E, Bellet M, Bonnefoi HR, Climent MA, Da Prada GA, Burstein HJ *et al*: **Adjuvant ovarian suppression in premenopausal breast cancer**. *The New England journal of medicine* 2015, **372**(5):436-446.
39. Pagani O, Regan MM, Walley BA, Fleming GF, Colleoni M, Lang I, Gomez HL, Tondini C, Burstein HJ, Perez EA *et al*: **Adjuvant exemestane with ovarian suppression in premenopausal breast cancer**. *The New England journal of medicine* 2014, **371**(2):107-118.
40. **https://nbcg.no/**
41. Early Breast Cancer Trialists' Collaborative G: **Comparisons between different polychemotherapy regimens for early breast cancer: meta-analyses of long-term outcome among 100 000 women in 123 randomised trials**. *Lancet (London, England)* 2012, **379**(9814):432-444.
42. Wishart GC, Bajdik CD, Dicks E, Provenzano E, Schmidt MK, Sherman M, Greenberg DC, Green AR, Gelmon KA, Kosma VM *et al*: **PREDICT Plus: development and validation of a prognostic model for early breast cancer that includes HER2**. *British journal of cancer* 2012, **107**(5):800-807.
43. Olivotto IA, Bajdik CD, Ravdin PM, Speers CH, Coldman AJ, Norris BD, Davis GJ, Chia SK, Gelmon KA: **Population-based validation of the prognostic model ADJUVANT! for early breast cancer**. *Journal of clinical oncology : official journal of the American Society of Clinical Oncology* 2005, **23**(12):2716-2725.
44. Perou CM, Sorlie T, Eisen MB, van de Rijn M, Jeffrey SS, Rees CA, Pollack JR, Ross DT, Johnsen H, Akslen LA *et al*: **Molecular portraits of human breast tumours**. *Nature* 2000, **406**(6797):747-752.
45. Sorlie T, Perou CM, Tibshirani R, Aas T, Geisler S, Johnsen H, Hastie T, Eisen MB, van de Rijn M, Jeffrey SS *et al*: **Gene expression patterns of breast carcinomas distinguish tumor subclasses with clinical implications**. *Proceedings of the National Academy of Sciences of the United States of America* 2001, **98**(19):10869-10874.
46. Farmer P, Bonnefoi H, Becette V, Tubiana-Hulin M, Fumoleau P, Larsimont D, Macgrogan G, Bergh J, Cameron D, Goldstein D *et al*: **Identification of molecular apocrine breast tumours by microarray analysis**. *Oncogene* 2005, **24**(29):4660-4671.
47. Herschkowitz JI, Simin K, Weigman VJ, Mikaelian I, Usary J, Hu Z, Rasmussen KE, Jones LP, Assefnia S, Chandrasekharan S *et al*: **Identification of conserved gene expression features between murine mammary carcinoma models and human breast tumors**. *Genome biology* 2007, **8**(5):R76.
48. Parker JS, Mullins M, Cheang MC, Leung S, Voduc D, Vickery T, Davies S, Fauron C, He X, Hu Z *et al*: **Supervised risk predictor of breast cancer based on intrinsic subtypes**. *Journal of clinical oncology : official journal of the American Society of Clinical Oncology* 2009, **27**(8):1160-1167.

49. Geiss GK, Bumgarner RE, Birditt B, Dahl T, Dowidar N, Dunaway DL, Fell HP, Ferree S, George RD, Grogan T *et al*: **Direct multiplexed measurement of gene expression with color-coded probe pairs**. *Nature biotechnology* 2008, **26**(3):317-325.
50. Sparano JA, Gray RJ, Makower DF, Pritchard KI, Albain KS, Hayes DF, Geyer CE, Jr., Dees EC, Perez EA, Olson JA, Jr. *et al*: **Prospective Validation of a 21-Gene Expression Assay in Breast Cancer**. *The New England journal of medicine* 2015, **373**(21):2005-2014.
51. Dubsky P, Brase JC, Jakesz R, Rudas M, Singer CF, Greil R, Dietze O, Luisser I, Klug E, Sedivy R *et al*: **The EndoPredict score provides prognostic information on late distant metastases in ER+/HER2- breast cancer patients**. *British journal of cancer* 2013, **109**(12):2959-2964.
52. Gnant M, Filipits M, Greil R, Stoeger H, Rudas M, Bago-Horvath Z, Mlineritsch B, Kwasny W, Knauer M, Singer C *et al*: **Predicting distant recurrence in receptor-positive breast cancer patients with limited clinicopathological risk: using the PAM50 Risk of Recurrence score in 1478 postmenopausal patients of the ABCSG-8 trial treated with adjuvant endocrine therapy alone**. *Annals of oncology : official journal of the European Society for Medical Oncology* 2014, **25**(2):339-345.
53. Sotiriou C, Pusztai L: **Gene-expression signatures in breast cancer**. *The New England journal of medicine* 2009, **360**(8):790-800.
54. van 't Veer LJ, Dai H, van de Vijver MJ, He YD, Hart AA, Mao M, Peterse HL, van der Kooy K, Marton MJ, Witteveen AT *et al*: **Gene expression profiling predicts clinical outcome of breast cancer**. *Nature* 2002, **415**(6871):530-536.
55. Foekens JA, Atkins D, Zhang Y, Sweep FC, Harbeck N, Paradiso A, Cufer T, Sieuwerts AM, Talantov D, Span PN *et al*: **Multicenter validation of a gene expression-based prognostic signature in lymph node-negative primary breast cancer**. *Journal of clinical oncology : official journal of the American Society of Clinical Oncology* 2006, **24**(11):1665-1671.
56. Paik S, Shak S, Tang G, Kim C, Baker J, Cronin M, Baehner FL, Walker MG, Watson D, Park T *et al*: **A multigene assay to predict recurrence of tamoxifen-treated, node-negative breast cancer**. *The New England journal of medicine* 2004, **351**(27):2817-2826.
57. Ward S, Scope A, Rafia R, Pandor A, Harnan S, Evans P, Wyld L: **Gene expression profiling and expanded immunohistochemistry tests to guide the use of adjuvant chemotherapy in breast cancer management: a systematic review and cost-effectiveness analysis**. *Health technology assessment (Winchester, England)* 2013, **17**(44):1-302.
58. Tang G, Cuzick J, Costantino JP, Dowsett M, Forbes JF, Crager M, Mamounas EP, Shak S, Wolmark N: **Risk of recurrence and chemotherapy benefit for patients with node-negative, estrogen receptor-positive breast cancer: recurrence score alone and integrated with pathologic and clinical factors**. *Journal of clinical oncology : official journal of the American Society of Clinical Oncology* 2011, **29**(33):4365-4372.

59. Sparano JA, Gray RJ, Makower DF, Pritchard KI, Albain KS, Hayes DF, Geyer CE, Jr., Dees EC, Goetz MP, Olson JA, Jr. *et al*: **Adjuvant Chemotherapy Guided by a 21-Gene Expression Assay in Breast Cancer**. *The New England journal of medicine* 2018, **379**(2):111-121.
60. Goncalves R, Bose R: **Using multigene tests to select treatment for early-stage breast cancer**. *Journal of the National Comprehensive Cancer Network : JNCCN* 2013, **11**(2):174-182; quiz 182.
61. **http://clinicaltrials.gov/show/NCT00310180**
62. Ronneberg JA, Fleischer T, Solvang HK, Nordgard SH, Edvardsen H, Potapenko I, Nebdal D, Daviaud C, Gut I, Bukholm I *et al*: **Methylation profiling with a panel of cancer related genes: association with estrogen receptor, TP53 mutation status and expression subtypes in sporadic breast cancer**. *Molecular oncology* 2011, **5**(1):61-76.
63. Rouzier R, Perou CM, Symmans WF, Ibrahim N, Cristofanilli M, Anderson K, Hess KR, Stec J, Ayers M, Wagner P *et al*: **Breast cancer molecular subtypes respond differently to preoperative chemotherapy**. *Clinical cancer research : an official journal of the American Association for Cancer Research* 2005, **11**(16):5678-5685.
64. Esserman LJ, Berry DA, Cheang MC, Yau C, Perou CM, Carey L, DeMichele A, Gray JW, Conway-Dorsey K, Lenburg ME *et al*: **Chemotherapy response and recurrence-free survival in neoadjuvant breast cancer depends on biomarker profiles: results from the I-SPY 1 TRIAL (CALGB 150007/150012; ACRIN 6657)**. *Breast cancer research and treatment* 2012, **132**(3):1049-1062.
65. Prat A, Bianchini G, Thomas M, Belousov A, Cheang MC, Koehler A, Gomez P, Semiglazov V, Eiermann W, Tjulandin S *et al*: **Research-based PAM50 subtype predictor identifies higher responses and improved survival outcomes in HER2-positive breast cancer in the NOAH study**. *Clinical cancer research : an official journal of the American Association for Cancer Research* 2014, **20**(2):511-521.
66. Filipits M, Nielsen TO, Rudas M, Greil R, Stoger H, Jakesz R, Bago-Horvath Z, Dietze O, Regitnig P, Gruber-Rossipal C *et al*: **The PAM50 risk-of-recurrence score predicts risk for late distant recurrence after endocrine therapy in postmenopausal women with endocrine-responsive early breast cancer**. *Clinical cancer research : an official journal of the American Association for Cancer Research* 2014, **20**(5):1298-1305.
67. Delahaye L, Drukker CA, Dreezen C, Witteveen A, Chan B, Snel M, Beumer IJ, Bernards R, Audeh MW, Van't Veer LJ *et al*: **A breast cancer gene signature for indolent disease**. *Breast cancer research and treatment* 2017, **164**(2):461-466.
68. Abrahams HJ, Gielissen MF, Schmits IC, Verhagen CA, Rovers MM, Knoop H: **Risk factors, prevalence, and course of severe fatigue after breast cancer treatment: a meta-analysis involving 12 327 breast cancer survivors**. *Annals of oncology : official journal of the European Society for Medical Oncology* 2016, **27**(6):965-974.

69. Reinertsen KV, Engebraaten O, Loge JH, Cvancarova M, Naume B, Wist E, Edvardsen H, Wille E, Bjoro T, Kiserud CE: **Fatigue During and After Breast Cancer Therapy-A Prospective Study**. *Journal of pain and symptom management* 2017, **53**(3):551-560.
70. Bardwell WA, Ancoli-Israel S: **Breast Cancer and Fatigue**. *Sleep medicine clinics* 2008, **3**(1):61-71.
71. Lænkholm A-V, Jensen M-B, Eriksen JO, Kibøll T, Rasmussen BB, Knoop AS, Ferree S, Haffner T, Schaper C, Ejlertsen B *et al*: **Prediction of 10yr distant recurrence (DR) using the Prosigna (PAM50) assay in a Danish Breast Cancer Cooperative Group (DBCG) cohort of postmenopausal Danish women with hormone receptor-positive (HR+) early breast cancer (EBC) allocated to 5yr of endocrine therapy (ET) alone**. *Journal of Clinical Oncology* 2015, **33**(15_suppl):546-546.
72. Ohnstad HO BE, Falk RS, Lien TG, Aaserud M, Sveli MAT, Kyte JA, Kristensen V, Geitvik G, Schlichting E, Wist E, Sørlie T, Russnes HG, Naume B: **Prognostic value of PAM50 and risk of recurrence score in patients with early stage breast cancer with long-term follow-up**. In. Edited by Hospital OU. Breast Cancer Research; 2017.
73. Anne Kuijer MS, Bianca den Dekker, Annelotte C.M. van Bommel, Sjoerd G. Elias, Carolien H., Smorenburg JW, Sabine C. Linn, Emiel J.Th. Rutgers, Sabine Siesling, and Thijs van Dalen: **Impact of 70-Gene Signature Use on Adjuvant Chemotherapy Decisions in Patients With Estrogen Receptor–Positive Early Breast Cancer: Results of a Prospective Cohort Study**. *Journal of Clinical Oncology* 2017.
74. Dowsett M, Cuzick J, Wale C, Forbes J, Mallon EA, Salter J, Quinn E, Dunbier A, Baum M, Buzdar A *et al*: **Prediction of risk of distant recurrence using the 21-gene recurrence score in node-negative and node-positive postmenopausal patients with breast cancer treated with anastrozole or tamoxifen: a TransATAC study**. *Journal of clinical oncology : official journal of the American Society of Clinical Oncology* 2010, **28**(11):1829-1834.
75. Wiedswang G, Borgen E, Karesen R, Kvalheim G, Nesland JM, Qvist H, Schlichting E, Sauer T, Janbu J, Harbitz T *et al*: **Detection of isolated tumor cells in bone marrow is an independent prognostic factor in breast cancer**. *Journal of clinical oncology : official journal of the American Society of Clinical Oncology* 2003, **21**(18):3469-3478.
76. NICE NIfHaCE: **Tumour profiling tests to guide adjuvant chemotherapy decisions in early breast cancer**. In.; 2018.
77. Chalder T, Berelowitz G, Pawlikowska T, Watts L, Wessely S, Wright D, Wallace EP: **Development of a fatigue scale**. *Journal of psychosomatic research* 1993, **37**(2):147-153.
78. Zigmond AS, Snaith RP: **The hospital anxiety and depression scale**. *Acta psychiatrica Scandinavica* 1983, **67**(6):361-370.

79. Elston CW, Ellis IO: **Pathological prognostic factors in breast cancer. I. The value of histological grade in breast cancer: experience from a large study with long-term follow-up**. *Histopathology* 1991, **19**(5):403-410.
80. Peto R, Davies C, Godwin J, Gray R, Pan HC, Clarke M, Cutter D, Darby S, McGale P, Taylor C *et al*: **Comparisons between different polychemotherapy regimens for early breast cancer: meta-analyses of long-term outcome among 100,000 women in 123 randomised trials**. *Lancet (London, England)* 2012, **379**(9814):432-444.

10. Appendix 1: Participating hospitals

The following hospitals have confirmed participation:

Date: 05.05.21

Oslo University Hospital

Akershus University Hospital

Østfold Hospital

Vestre Viken Hospital

Vestfold Hospital

Telemark Hospital

Haukeland University Hospital

Stavanger University Hospital

St. Olavs Hospital

Molde Hospital

Ålesund Hospital

University Hospital of North Norway

Nordland Hospital Bodø

Sørlandet Hospital

Innlandet Hospital

Førde Hospital

Haugesund Hospital

Levanger Hospital

11. Appendix 2: Protocol history

Version 1.0:

Version	Version date	Date Submitted to REC	Submission	REC opinion	Comments
V1.0	25 Oct 2017	31 Oct 2017	Initial application	Approved 20 Dec 2017	n/a

Version 2.0:

<table>
<tr><th>Version</th><th>Version date</th><th>Date Submitted to REC</th><th>Submission</th><th>REC opinion</th><th>Comments</th></tr>
<tr><td>V2.0</td><td>10 Apr 2019</td><td>23 Apr 2019</td><td>SA#1</td><td>Approved 07 Jun 2019</td><td>n/a</td></tr>
<tr><td colspan="6"><u>Summary of changes made in V2.0:</u>
• Minor text changes for clarification purposes.
• Updating of study background and rationale with recent relevant evidence
• Clarification of inclusion criteria with removal of PgR status since ER status is the lead marker. ER positive defined as ≥ 1%.
• Relocating the Prosigna test in the study design (Figure 3) so that the test is scheduled <u>after</u> first postoperative visit to promote treatment decision both with and without the test-result.
• Addition of patient questionnaires at 3 and 6 months after study inclusion to receive information about early side effects.
• Addition of patient questionnaire FACT-ES to register endocrine symptoms thoroughly.
• Replace the patient questionnaire SF-36 with RAND-36 that includes almost identical questions, but RAND-36 is also in use for the general Norwegian population as standard of reference.
• The patients questionnaire HADS-A is replaced with GAD-7 due to economical conditions.
• Update section 7.2 Statistical consideration since determination of breast cancer specific survival and metastasis free survival by error were missing in protocol v1.0</td></tr>
<tr><td colspan="6">Number of patients included when V2.0 approved: 82</td></tr>
</table>

Version 2.1:

Version	Version date	Date Submitted to REC	Submission	REC opinion	Comments
V2.0	14 Oct 2019	04 Nov 2019	SA#2	Approved 06 Dec 2019	n/a

Summary of changes made in V2.1:
• Clarification of completed surgery in the inclusion criteria (re-resection is permitted given no change in tumor size categorization is expected)<br>• Adjustment in the study design (Figure 3) due to patient logistics: The Prosigna test is scheduled after first postoperative visit <u>or</u> postoperative multidisciplinary tumor board meeting if postoperative visit is not part of the regular visits at the treating hospital.<br>• Introduction of a procedure for remote (verbal) consent.
Number of patients included when V2.1 approved: 170

Version 2.2:

Version	Version date	Date Submitted to REC	Submission	REC opinion	Comments
V2.2	23 Jun 2020	26 Jun 2020	SA#3	Approved 30 Jun 2020	n/a
<u>Summary of changes made in V2.2:</u><br>• Correction of small textual changes and references included in V2.0 that erroneously was omitted in V2.1.					
Number of patients included when V2.2 approved: 443					

Version 3.0:

Version	Version date	Date Submitted to REC	Submission	REC opinion	Comments
V3.0	22 Sep 2020	30 Sep 2020	SA#4	Approved 19 Oct 2020	n/a
<u>Summary of changes made in V3.0:</u><br>• Expanding the study population to include also patients with lymph node micrometastases if tumor size is ≤ 20mm to make the Prosigna test available for this subgroup of patients clinically treated as lymph node negative in line with the assumption that the prognosis is more similar to patients with lymph node negative status than those with more widespread lymph node positive disease. Including the patients in the study enables information retrieval and follow-up. The additional number of patients in this group is expected to be limited, and the inclusion period and number of lymph node negative patients to be included will be unchanged. Consequently, the protocol change will not affect the power of the study. The lymph node micrometastasis subgroup will be studied by explorative analyses; this group will not be included in the survival analysis due to uncertainty about how similar the prognosis for this subgroup is compared to the lymph node negative subgroup.<br>• Patients are allowed to participate in concurrent clinical studies which do not affect the main endpoints of this study. If included in such studies, these patients will be "flagged" to evaluate their impact on the secondary endpoints in the present study.<br>• Oslo2 study specific details relevant for patients included in both studies (i.e. EMIT-1 and Oslo2) are removed due to recruitment halt in the Oslo2 study.					
Number of patients included when V3.0 approved: 586					

Version 3.1:

Version	Version date	Date Submitted to REC	Submission	REC opinion	Comments
V3.1	5 May 2021	20 May 2021	SA#5	Approved 11 Jun 2021	
Summary of changes made in V3.1:<br>• Updated participating study hospitals (appendix 1) and the corresponding organization overview (Figure 2).<br>• Correction of the exclusion criteria in §5.3, to comply with corresponding criteria in the Study Synopsis (section 1). The correction in §5.3 was erroneously omitted in V3.0.<br>• A new layout of the study schematic overview is included for clarification.<br>• The follow-up plan (section 4.2 and 6.2) is defined more precisely.<br>• Minor text changes for clarification purposes.					
Number of patients included when V3.1 approved: 1178					

Version 3.2

Version	Version date	Date Submitted to REC	Submission	REC opinion	Comments
V3.2	09 May 2023	15 May 2023	SA#6	Approved 25 May 2023	
Summary of changes made in V3.2:<br>• The Norwegian database for sick leaves and occupational disability is no longer located in FD Trygd, but in SSB (Statistics Norway), hence updated.<br>• Updated endpoint nomenclature according to published Standardized Definitions for Efficacy End Points (STEEP) in Adjuvant Breast Cancer Clinical Trials, STEEP v2.0 and adjusting relevant statistical analysis accordingly. No change in the study aims.					
Number of patients included when V3.2 approved: 2320					
